# Single intracerebroventricular TNFR2 agonist injection impacts remyelination in the cuprizone model

**DOI:** 10.1007/s00109-025-02549-6

**Published:** 2025-05-10

**Authors:** Valentina Pegoretti, Ate Boerema, Kim Kats, Juan M. Dafauce Garcia, Roman Fischer, Roland E. Kontermann, Klaus Pfizenmaier, Jon D. Laman, Ulrich L. M. Eisel, Wia Baron

**Affiliations:** 1 Department of Molecular Neurobiology, Groningen, Institute of Evolutionary Life Science (GELIFES), University of Groningen, Groningen, The Netherlands; 2 Department Biomedical Sciences, Section Molecular Cell Biology, University of Groningen, University Medical Center Groningen (UMCG), Groningen, The Netherlands; 3 Institute of Cell Biology and Immunology, University of Stuttgart, Germany; Stuttgart Research Centre Systems Biology, University of Stuttgart, Stuttgart, Germany; 4 Department Pathology and Medical Biology, University of Groningen, University Medical Center Groningen (UMCG), Groningen, The Netherlands; 5 Department Biomedical Sciences, Section Molecular Neurobiology, University of Groningen, University Medical Center Groningen (UMCG), MS Center Noord Nederland (MSCNN), A. Deusinglaan 1, 9713 AV Groningen, The Netherlands

**Keywords:** Cuprizone model, Multiple sclerosis, Nanotomy, Oligodendrocyte lineage cells, Remyelination, Tumor necrosis factor receptor 2

## Abstract

**Abstract:**

The development of therapeutics that enhances the regeneration of myelin sheaths following demyelination is predicted to prevent neurodegeneration. A promising target to enhance remyelination is the immunomodulatory cytokine tumor necrosis factor alpha (TNFα) and its receptors TNFR1 and TNFR2. TNFR2 on oligodendrocyte lineage cells and microglia coordinates different protective functions, such as proliferation of oligodendrocyte progenitor cells, survival of mature oligodendrocytes, and release of anti-inflammatory cytokines, in animal models of inflammation and demyelination. Here, we find in the cuprizone model that following demyelination, fewer axons are unmyelinated in the corpus callosum at an early stage of remyelination after single TNFR2 agonist delivery in the lateral ventricle, while astrocyte and microglia number and coverage are unchanged. Towards later stages of remyelination, TNFR2 agonist treatment maintains the number of oligodendrocyte lineage cells, and large caliber axons have thinner myelin. Hence, even short-term stimulation of TNFR2 has a positive impact on the remyelination processes. This study informs further on the beneficial implications of TNFR2 signaling on oligodendrocyte lineage cells and remyelination, emphasizing its potential therapeutic value for demyelinating diseases, including multiple sclerosis.

**Key messages:**

Single TNFR2 agonist treatment in the lateral ventricle following cuprizone-induced demyelination impacts remyelination by:

- Leading to a lower percentage of unmyelinated axons at early stages.
- Preserving the number of oligodendrocyte lineage cells in the corpus callosum at later stages.
- Covering large calibre axons with thinner myelin sheaths at later stages.

**Supplementary Information:**

The online version contains supplementary material available at 10.1007/s00109-025-02549-6.

## Introduction

Multiple sclerosis (MS) is a chronic demyelinating disease of the central nervous system (CNS). While the etiology of MS is not known, neuroinflammation and neurodegeneration are major hallmarks. Remyelination restores metabolic support to the axon and rapid signal transmission following myelin damage [1]. Efficient remyelination requires the activation of oligodendrocyte progenitor cells (OPC), their subsequent recruitment (proliferation and migration) to the demyelinated area followed by differentiation into mature myelinating oligodendrocytes (OLG) [2]. Failure of the remyelination process leads to secondary axonal degeneration and neuronal loss. Understanding the underlying mechanisms and possible drug targets that enhance remyelination at lesion sites has great therapeutic potential. Current treatments reduce relapses by dampening peripheral inflammation but do not trigger tissue repair [3]. Key mediators that enhance the ability of CNS to regenerate myelin sheaths are therefore needed.

Targeting receptors for tumor necrosis factor (TNF) is a novel strategy to modulate both peripheral inflammation and remyelination in MS [4, 5]. TNF is an immunomodulatory cytokine with a broad range of activities [6, 7] acting on two distinct TNFα receptors (TNFR) displaying different expression patterns, intracellular signaling, and functions. Solube TNF (sTNF) binds with greater affinity to TNFR1 due to stronger stability of ligand-receptor complexes [8] and transmembrane TNF (tmTNF) preferentially stimulates TNFR2 [9]. In MS, non-selective inhibition of both sTNF and tmTNF with Lenercept was not beneficial [10]. On the other hand, treatment with a selective blocker of sTNF (XPro1595) reduced paralysis symptoms in experimental autoimmune encephalomyelitis (EAE), an autoimmune inflammation-mediated demyelination model for MS [11]. Furthermore, in two different EAE studies, TNFR1 inhibition required TNFR2 stimulation to exert robust beneficial effects on paralysis symptoms but also on autoimmune inflammation-induced demyelination [12, 13]. Hence, while pharmacological treatment blocking TNFR1 led to immune system suppression, a decrease in EAE symptoms and pathology [14], the role of TNFR2 in neuroimmune diseases is just beginning to be characterized.

By inhibiting sTNF, XPro1595 treatment beneficially affected multiple aspects of the remyelination process in EAE, such as axon preservation, myelin compaction, and an increased number of OPC, likely via enhanced TNFR2 signaling [11]. Fittingly, ablation of TNFR2 prevented remyelination in EAE, resulting in a higher severity of paralysis [15]. The beneficial effect of TNFR2 in EAE is driven to some extent by CNS-resident microglia and OLG. More specifically, conditional knock-out of TNFR2 in microglia led to early onset and increased T cell activation and demyelination, while ablation of TNFR2 in OLG led to impaired OPC differentiation and reduced remyelination [16]. Similarly, in the cuprizone mouse model for non-inflammatory-mediated demyelination, global ablation of TNFR2 delayed OPC proliferation, reduced OLG numbers, and hampered remyelination [17]. Moreover, peripheral treatment of cuprizone-fed mice with blood–brain barrier (BBB) permeable XPro1595 accelerated remyelination by increasing microglia-mediated myelin phagocytosis most likely via modulation of TNFR2 [18]. Taken together, pharmacological targeting by selective activation of TNF-TNFR2 signaling showed promising beneficial effects in terms of immune modulation and tissue repair, including remyelination [19, 20].

To further explore the therapeutic value of TNFR2 in demyelinating diseases, we investigated the impact of a single intracerebroventricular (i.c.v.) injection of human TNFR2 agonist EHD2-scTNF_R2_ into the brain on remyelination in the cuprizone mouse model following demyelination. By combining local administration in the brain and a model of de- and re-myelination with CNS origin, we aimed to minimize the peripheral contribution on the disease pathology as well as therapeutic effects thereby focusing on CNS mechanisms. Our findings revealed that, in an early remyelination stage, the percentage of unmyelinated axons was lower upon EHD2-scTNF_R2_ treatment following cuprizone-induced demyelination in the corpus callosum (CC) than upon saline treatment. Towards the later stages of remyelination, the number of oligodendrocyte lineage cells was maintained, while large axons were covered with thinner myelin sheaths compared to saline treatment. Hence, this study provides new insight into the therapeutic value of TNFR2 agonists in remyelination.

## Materials and methods

### Animals

Human/murine TNFR2 knock-in (B6.B6-huTNFRSF1B_ecd_^tm2UEG^, hu/m TNFR2-ki) mice were generated by Ozgene Pty Ltd. (Bentley, Australia) as described [21]. The extracellular domain of TNFR2 has been replaced by the human counterpart, while the intracellular part remains murine. Both mouse TNF and human TNF can interact with and activate the chimeric receptor. The expression pattern and level of the chimeric hu/mTNFR2 are comparable to wild-type mouse TNFR2 [21]. Animals were kept on a 12-h light/12-h dark schedule and had water and food ad libitum except during the cuprizone feeding phase. The care and treatment of the animals were carried out in accordance with the guidelines of the animal experimentation committee Groningen on the use of experimental animals at the University of Groningen (CCD no.: AVD105002016504; IvD no.: 1650404-02).

### Cuprizone model and treatment

Eight-week-old hu/m TNFR2-ki male mice were fed with 0.2% cuprizone diet. The hu/m TNFR2-ki mice have a C57BL/6 genetic background, which is widely used in the cuprizone model [22, 23]. After 5 weeks of cuprizone feeding, the animals were treated with a single intracerebroventricular (ICV; coordinates: − 0.05 anteroposterior; − 0.1 medial lateral; − 0.25 dorsal ventral) injection of a human TNFR2 agonist compound (EHD2-scTNF_R2_; 10 µg in 6.25 µl PBS). The selectivity of EHD2-scTNF_R2_ was validated by binding studies, and the molecule was tested previously in hu/m TNFR2-ki mice [12, 21]. After the injection, mice were fed with standard chow. Animals were transcardially perfused, and tissue was collected at 7- or 14-days post treatment (DPT). The detailed procedures are provided in the Supplementary Materials and Methods.

### Experimental assays

To assess the extent of remyelination in the corpus callosum (CC) upon saline and EHD2-scTNF_R2_ treatment, we analyzed myelin density using Black Gold II and MBP, along with ultrastructural parameters of individual (myelinated) axons, such as g-ratio, axon radius, myelin thickness, and compaction using large 2D scale scanning transmission electron microscopy (STEM). In addition, immunohistochemical analyses were conducted to examine microglia (IBA1), astrocytes (GFAP), and oligodendrocyte lineage cells (Olig2). The in vivo findings were complemented by exploring the effect of EHD2-scTNF_R2_ on microglia activation and OPC proliferation in vitro. Specific protocols for each assay are outlined in the Supplementary Materials and Methods.

### Statistical analysis

Data are presented as mean standard error of mean (SEM). The Shapiro–Wilk test was used to test the normal distribution of the data. Statistical analyses were performed by unpaired *t*-test to compare two treatment groups and/or two time points. When analyzing differences in percentage, the nonparametric Mann–Whitney *U* test was used. The correlation between axon diameter and myelin thickness was tested with the Pearson coefficient, while differences in the degree of myelin compaction were tested with a general linear multivariate model. Body weight change over time was tested with two-way ANOVA (or mixed model) with Sidak’s multiple comparison test to compare between treatments at each time point. qPCR and in vitro data were analyzed using one-sample *t*-test with vehicle (PBS)-treated samples set at 1 in each independent cell culture experiment. A value of *p* < 0.05 was considered statistically significant. Statistics and data plotting were performed with IBM SPSS Statistics v26 and GraphPad Prism Software v8 (San Diego, California).

## Results

### Single TNFR2 agonist treatment following cuprizone-induced demyelination results in a lower percentage of unmyelinated axons during the early remyelination stage

**Fig. 1:**
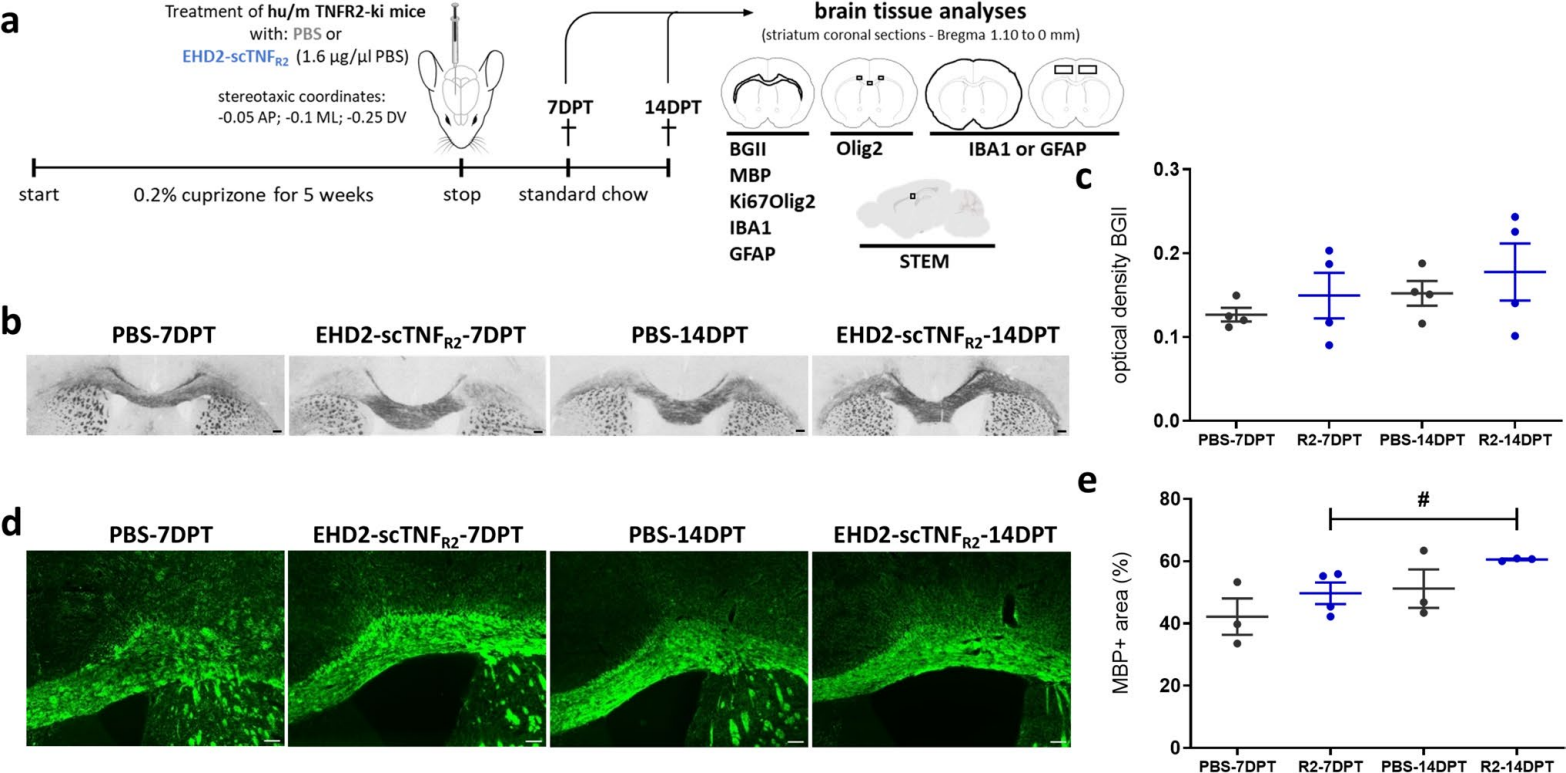
Single EHD2-scTNF_R2_ treatment following cuprizone-induced demyelination increases myelin density in the corpus callosum over time. **a** Schematic representation of the experimental design and analyses. To induce demyelination, hu/m TNFR2 knock-in mice were fed for 5 weeks with 0.2% cuprizone. At 5 weeks of cuprizone feeding, mice were injected intracerebroventricularly (− 0.05 anteroposterior: AP; − 0.1 medial lateral: ML; − 0.25 dorsal ventral: DV) with saline (PBS, grey) or the human TNFR2 agonist EHD2-scTNF_R2_ (R2, blue), fed with normal chow and analyzed 7 or 14 days (DPT) post treatment (DPT). To visualize myelin, coronal brain sections (Bregma 1.10 mm to 0 mm) were stained with Black Gold II (BGII, **b**) and for myelin basic protein (MBP, **d**). The optical density of BGII signal (**c**) and the percentage of MBP coverage (**e**) in the corpus callosum (CC) were measured. *n* = *3–4 animals/group, An unpaired t-test (****c***) *or Mann–Whitney U test (****e****) were used to compare between two treatment groups at the same time point (****c***, ***e***, not significant), or two time points of the same treatment (***c***, ***e***, #p < *0.05). Scale bars are 100 µm. a was created using vectors from scidraw.io*

A frequently used model for remyelination research is the dietary cuprizone model (Fig. 1a). The copper chelator cuprizone induces loss of mature OLG, leading to global and maximum demyelination in the CC at 5 weeks cuprizone intoxication. In the CC, OPC activation and recruitment, important for the onset of the remyelination process, typically begins between the third and fifth week of cuprizone feeding. Mature OLG and remyelinated axons are observed within 1 week, and robust remyelination is achieved within 2 weeks after cuprizone withdrawal [22, 24–26]. To determine whether TNFR2 stimulation impacts remyelination, saline (PBS) or the TNFR2 agonist EHD2-scTNF_R2_ were delivered into the brain following 5-week cuprizone-induced demyelination by a single i.c.v. injection (Fig. 1a). Treating early would primarily address effects on demyelination. Since EHD2-scTNF_R2_ is a human TNFR2 agonist, we used chimeric hu/m TNFR2 knock-in mice, in which the extracellular TNFR2 domain is human and the intracellular domain mouse, maintaining normal mouse TNF signaling and ensuring model suitability [21].

To evaluate differences between treatments and the impact of the treatment over time, we examined effects after 1 week (7 days post treatment—DPT) and 2 weeks (14 DPT) after cuprizone removal. To assess the extent of myelin regeneration, the density of myelin in the CC was visualized with a gold-phosphate complex called Black Gold II (BGII) and immunohistochemistry for a major myelin protein, MBP. The optical density of BGII (Fig. 1b,c) and the percentage of MBP coverage (Fig. 1d,e) were slightly, but not significantly, increased at 14 days compared to 7 days post saline treatment, implying ongoing regeneration of myelin between 7 and 14 DPT. Similarly, myelin density increased between 7 and 14 DPT upon EHD2-scTNF_R2_ treatment, which was significant for MBP coverage (Fig. 1c, e). At both 7 and 14 DPT, the myelin density upon EHD2-scTNF_R2_ treatment was approximately 10–20% higher than upon PBS treatment, although the difference at each time point did not reach significance (Fig. 1c, e). 

**Fig. 2:**
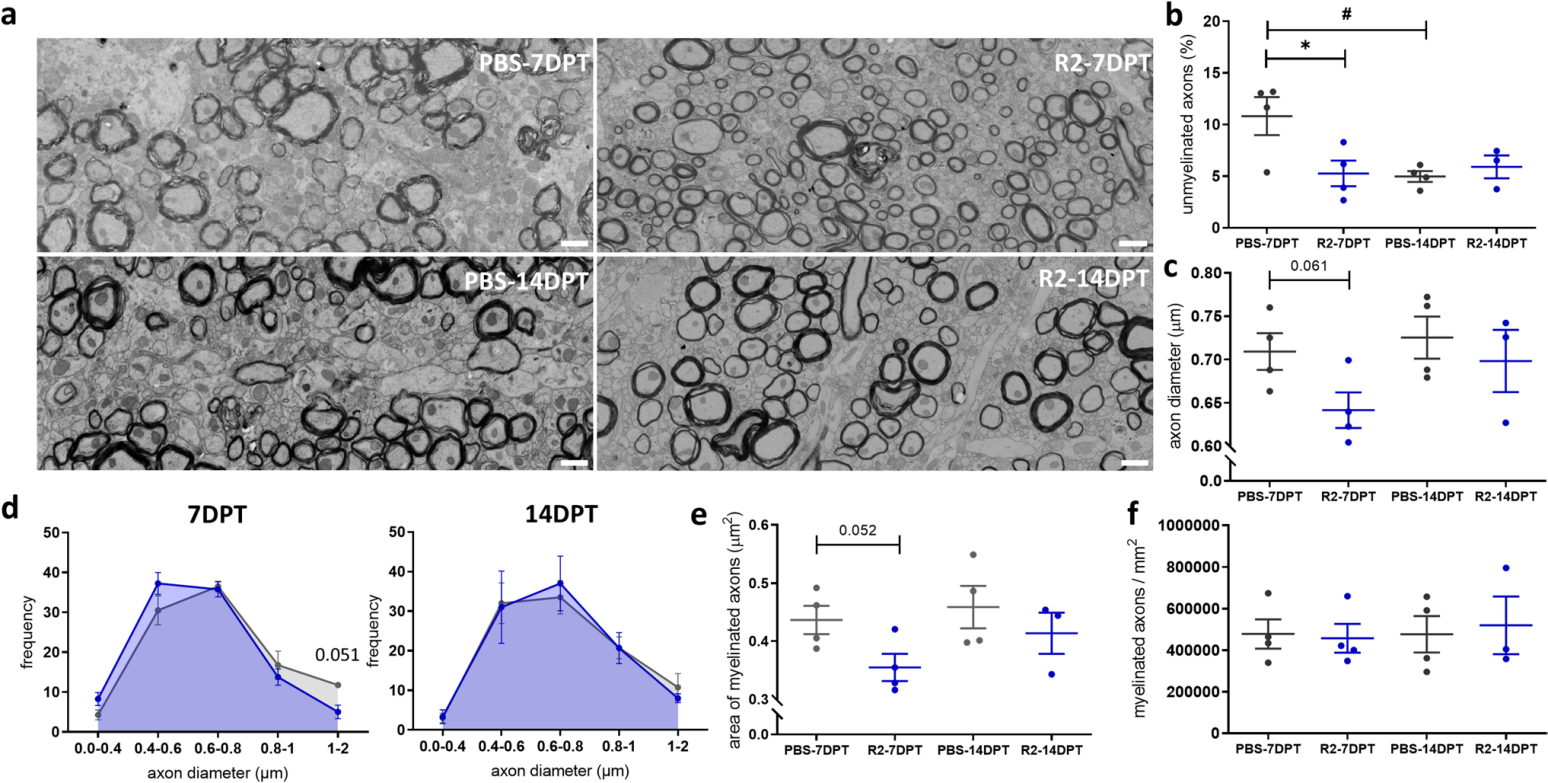
Single EHD2-scTNF_R2_ treatment following cuprizone-induced demyelination leads to a lower percentage of unmyelinated axons at 7 days post treatment. **a** Representative STEM images of the corpus callosum of mice treated either with saline (PBS, grey) or EHD2-scTNF_R2_ (R2, blue) 7 or 14 days (DPT) post treatment (DPT). STEM images are available at full resolution and scale at nanotomy.org. The percentage of unmyelinated axons of total axons (**b**), axon diameter (**c**), and myelin thickness were manually measured. The frequency of myelinated axons with different diameters (**d**) and average myelinated axon area per µm^2^ (**e**) were calculated*,* and myelinated axons per mm^2^ (**f**) counted. At least 100 axons per animal were manually measured. n = 3–4 animals/group. An unpaired t-test was conducted to compare between two treatments at the same time point (b, *p < 0.05) or between two time points of the same treatment (b, #p < 0.05). Scale bars are 1 µm

To more accurately assess remyelination, ultrastructural parameters of individual (myelinated) axons in large-scale STEM images were analyzed next (Fig. 2a, Table S3). To compare the level of remyelination, we first counted the number of denuded and myelinated axons. At 7 DPT, the percentage of unmyelinated axons was lower in EHD2-scTNF_R2_-treated animals compared to saline treatment, and similar to 14 DPT (Fig. 2b). At 14 DPT, both upon saline and EHD2-scTNF_R2_ treatment, approximately 5% of the axons were unmyelinated. Next, we measured the axon diameter of myelinated axons and observed that, at 7 DPT, the average axon diameter was about 70 nm smaller in EHD2-scTNF_R2_-treated animals without reaching significance, while, at 14 DPT, axon diameter was similar between treatments (Fig. 2c). In comparison with saline, EHD2-scTNF_R2_ treatment shifted the axon size distribution towards smaller caliber axons at 7 DPT, but not at 14 DPT (Fig. 2d). Furthermore, the myelinated axon area was less following EHD2-scTNF_R2_ treatment, especially at 7 DPT (Fig. 2e, p = 0.052), while the number of myelinated axons was comparable (Fig. 2f). As changes in axonal size may affect mitochondrial distribution and area [27], we analyzed the number and area of mitochondria in myelinated axons at 7 DPT. In the cross-sectional images, mitochondria in myelinated axons tended to have a larger area upon saline treatment than upon EHD2-scTNF_R2_ treatment. Conversely, there were no changes in mitochondrial content (% axon area), number of mitochondria per myelinated axon, or percentage of axons with mitochondria between treatments at 7 DPT (Fig. S3a-c). These findings suggest that compared to saline treatment, EHD2-scTNF_R2_ treatment accelerated the reduction in unmyelinated axons compared to saline treatment, while during early remyelination myelinated axons appear transiently smaller.

### Single TNFR2 agonist treatment following cuprizone-induced demyelination results in thinner myelin sheaths

**Fig. 3:**
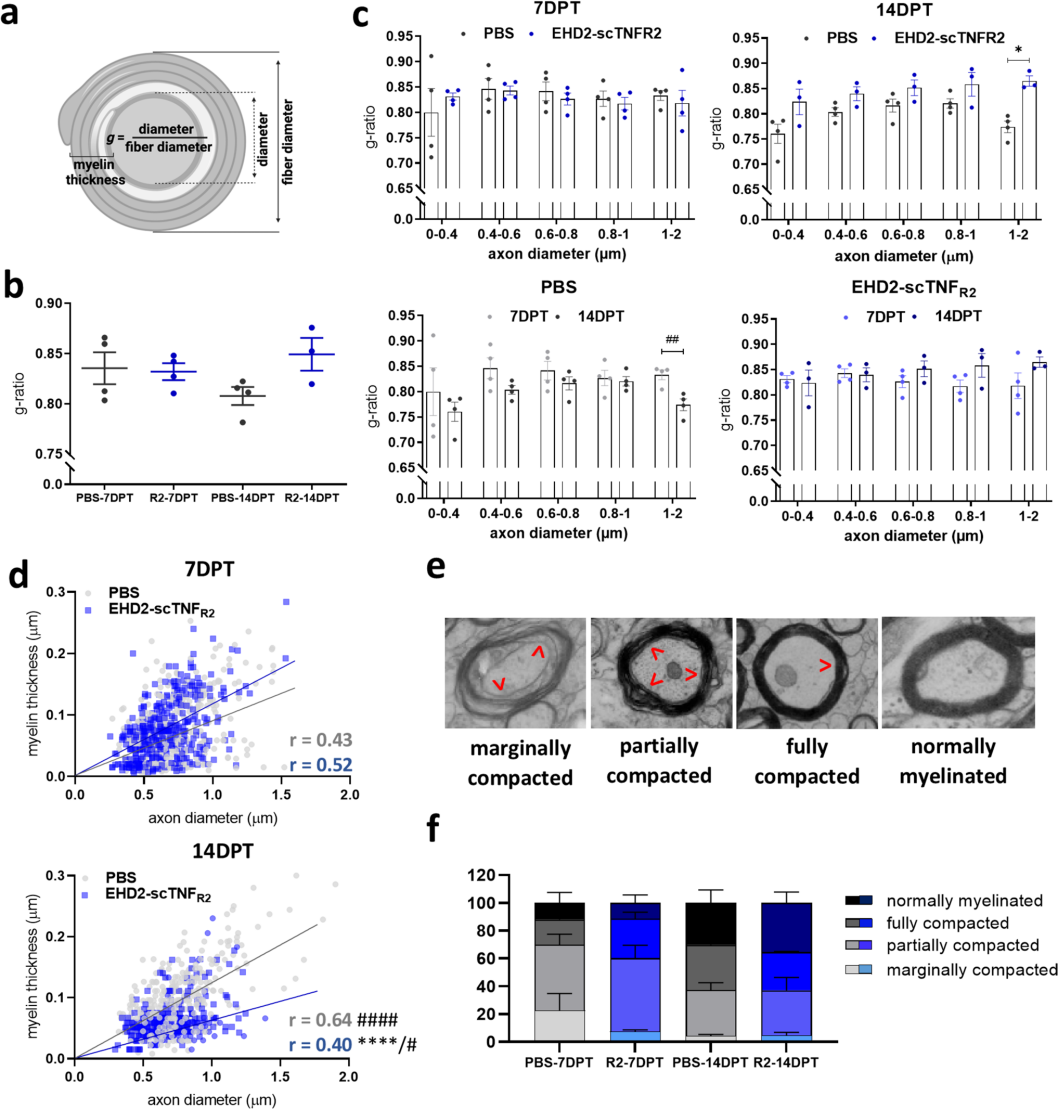
Single EHD2-scTNF_R2_ treatment following cuprizone-induced demyelination results in thinner myelin sheaths. STEM image analysis of the corpus callosum of mice treated either with saline (PBS, grey) or EHD2-scTNF_R2_ (R2, blue) 7 or 14 days (DPT) post treatment (DPT). From axon and fiber diameters (**a**), the g-ratio for each axon was calculated, and the g-ratio as average (**b**) and in relation to average axon diameter are plotted (**c**). For each animal, the individual myelinated axon diameter was plotted against its myelin thickness (**d**), highlighting changes in axon size and myelination over time. Solid lines represent the linear (**d**) regression line. Myelinated axons were scored on their degree of myelin compaction (**e,** red arrowheads point at non-compacted myelin) and plotted as percentage of the axons analyzed (**f**). At least 100 axons per animal were manually measured. *n* = *3–4 animals/group. An unpaired t-test was used to compare between two treatments at the same time point (****c****, ****d,*** *p < 0.05, ****p < *0.00005) or between two time points of the same treatment (***c***, ****d****, #p* < *0.05, ##p* < *0.01, ####p* < *0.00001). Pearson correlation was used to test the relationship between myelin thickness and diameter of each axon (***d***). Differences in the degree of myelin compaction were tested with a general linear multivariate model (***f***, not significant)*

A widely used index to assess myelin is the g-ratio, defined by the ratio between axon diameter and the myelinated fiber diameter (Fig. 3a, b, Table S3). A hallmark of remyelination is the presence of thinner myelin sheaths compared to myelin sheaths generated during developmental myelination [28, 29], resulting in a g-ratio above 0.8 [30]. At 7 and 14 DPT, both small and large caliber axons displayed an average g-ratio around and above 0.8, pointing to remyelinated axons [30].

At 14 DPT, but not 7 DPT, myelinated axons in EHD2-scTNF_R2_-treated animals tended to have a higher g-ratio than myelinated axons in saline-treated animals, which reached significance for large caliber axons (between 1 and 2 µm). Interestingly, in the saline-treated group, the g-ratio of large-caliber, and to a lesser extent, small-caliber, axons decreased over time, whereas this was not observed in the EHD2-scTNF_R2_ group (Fig. 3c), potentially reflecting differences in ongoing myelin membrane wrapping. Furthermore, myelin thickness and axon diameter of each animal correlated for both saline and EHD2-scTNF_R2_ treatment at 7 and 14 DPT. Correlation coefficients differed significantly between the two treatment groups at 14 DPT and between two time points of the same treatment (Fig. 3d). This further highlights that myelin of particularly larger axons at 14 DPT was thinner upon EHD2-scTNF_R2_ treatment.

To analyze a higher number of axons, we developed an algorithm which quantifies in STEM images the area covered by myelin and the axon size in an aggregated manner, thus without measuring the properties of each individual axon (Fig. S4a). In line with the manual analysis of individual myelinated axons, at 14 DPT, EHD2-scTNF_R2_-treated animals tended to have a higher aggregated g-ratio than saline-treated animals (Fig. S4b). Notably, the aggregated g-ratio and axon area values were lower than the manual analysis (Fig. S4b *vs* Fig. 3b and Fig. S4c *vs* Fig. 2e), and the aggregated axon area of myelinated axons did not differ between treatments and time points upon EHD2-scTNF_R2_ treatment (Fig. S4c). The percentage of myelinated area, referring to the area covered by myelin, increased between 7 and 14 DPT in saline-treated animals, whereas it decreased upon EHD2-scTNF_R2_ treatment (Fig. S4 d).

Thus, both manual and automated analyses signified that axons in EHD2-scTNF_R2_-treated animals have thinner myelin at 14 DPT. To assess whether myelin was thinner because of a difference in (de)compaction, we next assessed the degree of myelin compaction following a previously described scoring method (Fig. 3e) [31]. Both treatment groups showed similar levels of myelin compaction at 14 DPT (Fig. 3f). However, at 7 DPT, saline-treated animals had around 20% of their axons marginally compacted, while only around 7% of axons of animals treated with EHD2-scTNF_R2_ have marginally compacted myelin (Fig. 3f). Axons with fully and partially compacted myelin were more abundant in the EHD2-scTNF_R2_-treated animals at 7 DPT (Fig. 3f), pointing to more myelinated axons being in a later phase of the remyelination process. To assess whether the thinner myelin sheaths in EHD2-scTNF_R2_-treated animals at 14 DPT resulted from greater compaction, we acquired high-resolution STEM cross-sectional images of large axons at 14 DPT. While large myelinated axons in the EHD2-scTNF_R2_-treated group may have fewer myelin membrane layers, the myelin sheath compaction appeared more advanced, with clear inner tongues and a more distinct and abundant radial component (Fig. S5) [32]. Therefore, a single i.c.v. injection of a TNFR2 agonist following cuprizone-induced demyelination transiently shifted the myelinated axon size distribution towards smaller caliber axons at early stages of remyelination, which culminated in thinner regenerated myelin sheaths around large-caliber axons at a later stage.

### Single TNFR2 agonist treatment following cuprizone-induced demyelination does not induce major changes in microglia and astrocyte abundance in the corpus callosum

**Fig. 4:**
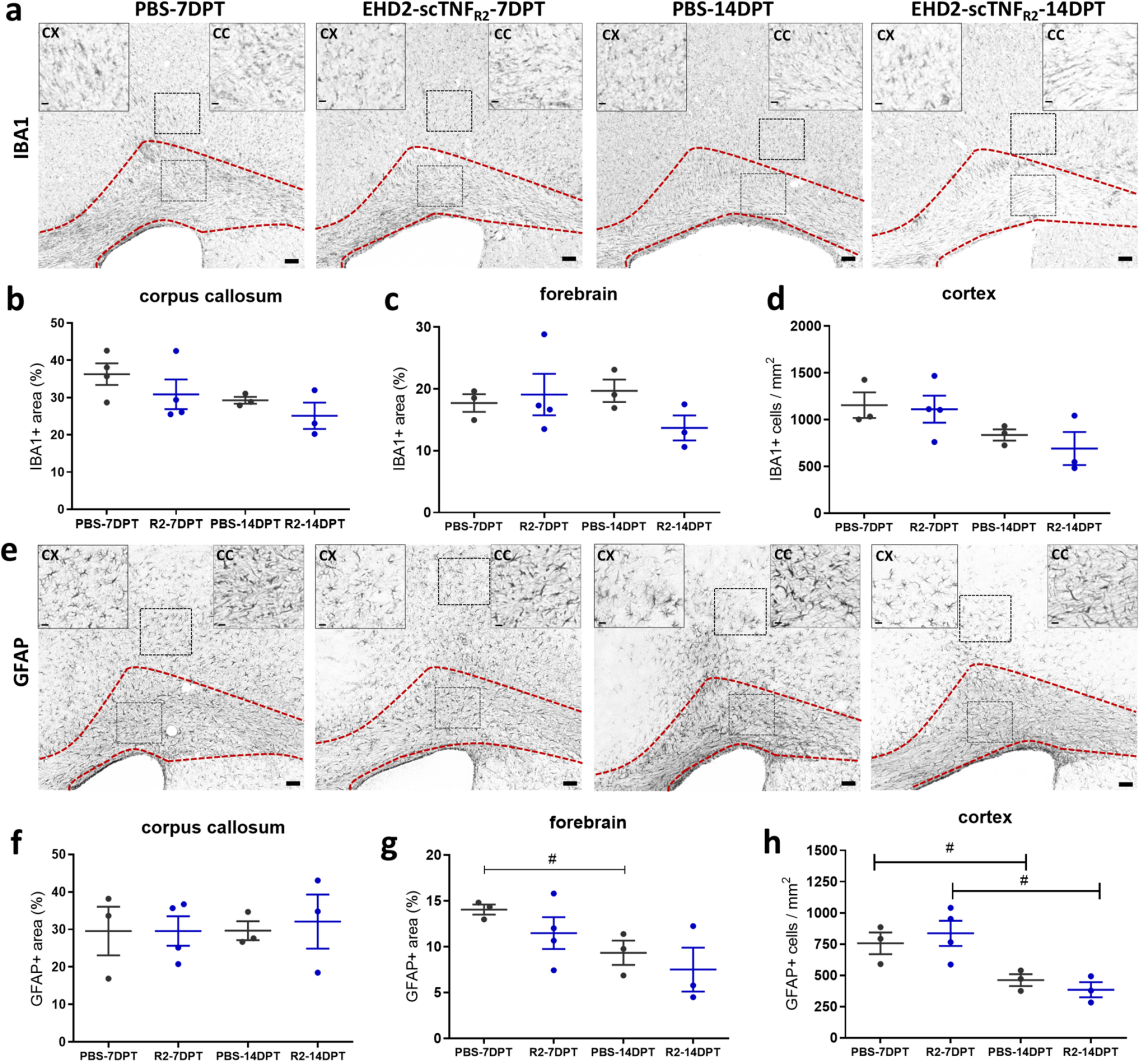
Single EHD2-scTNF_R2_ treatment following cuprizone-induced demyelination does not induce major changes in microglia and astrocyte abundance in the corpus callosum. Representative images of IBA1 (**a**, microglia marker) and GFAP staining (**e**, astrocyte marker) of the corpus callosum (CC, red dashed line) and whole forebrain of mice treated with saline (PBS, grey) or EHD2-scTNF_R2_ (R2, blue) and analyzed 7 or 14 days (DPT) post treatment (DPT). Coronal brain sections were analyzed for the coverage of IBA1 (**b**, **c**) or GFAP (**f**, **g**), while positive cells were counted in the cortex (CX; **d**, **h**). *n = 3-4 animals/group. An unpaired t-test (c) or Mann-Witney U test (e) were used to compare between two treatments at the same time point (b–d, f–h, not significant) or between two time points of the same treatment ****(b–d****, ****f–h,**** #p < 0.05). Scale bars are 100 µm (overview) and 10 µm (insets)*

Activation of microglia and astrocytes is a well-characterized hallmark of cuprizone-induced demyelination [24, 33, 34]. Microglial and astrocyte activation starts as early as 2 weeks of cuprizone feeding, and their activation sustains during remyelination [24]. Therefore, as activated astrocytes and microglia play a crucial role in facilitating remyelination [24] and express TNFR2 in the demyelinated CC [35, 36], we next assessed if the TNFR2 agonist affected the sustained neuroinflammatory response during the remyelination process following cuprizone-induced demyelination. We focused our analysis on the most affected area, the CC, and the whole forebrain, including cortical [37] and basal ganglia [38] regions, relevant areas for neuroinflammatory responses in the cuprizone model. In the CC, the coverage of IBA1-positive microglia notably, but not significantly, decreased between 7 and 14 DPT for both treatments (Fig. 4a, b). Compared to saline, EHD2-scTNF_R2_ treatment tended to reduce IBA1-positive microglia coverage at 14 DPT in the forebrain (Fig. 4a, c, p = 0.09) and led to a slight, but not significant, decrease in the number of IBA1-positive cells at 14 DPT in the cortex (Fig. 4a, d). Next, we quantified the mRNA expression of pro-inflammatory and anti-inflammatory genes in primary hu/m TNFR2-ki microglia treated with EHD2-scTNF_R2_ or PBS for 24 h (Fig. S6a). Expression of anti-inflammatory genes such as *Arg1* and *Il10* was upregulated upon TNFR2 agonist treatment compared to vehicle-treated microglia (Fig. S6c). On the other hand, EHD2-scTNF_R2_ treatment in vitro did not lead to changes in the expression of pro-inflammatory genes such as *Tnf, Il1b*, and *Nos2* (Fig. S6b).

The distribution of GFAP-positive astrocyte in the CC appeared unaffected (Fig. 4e, f), while the GFAP-positive astrocyte coverage in the forebrain decreased between 7 and 14 DPT (Fig. 4e, g). Similarly, the number of GFAP-positive cells in the cortex decreased for both saline and EHD2-scTNF_R2_ treatment between 7 and 14 DPT (Fig. 4e, h), indicating that the coverage is likely a reflection of the astrocyte number and not the number and length of their extensions. At both 7 and 14 DPT, no statistically significant difference was observed between treatments. Taken together, TNFR2 agonist treatment does not induce major changes in microglia and astrocyte abundance in the CC.

### Single TNFR2 agonist treatment following cuprizone-induced demyelination maintains oligodendrocyte lineage cell numbers

Remyelination following demyelination requires OPC recruitment, including proliferation and migration, and their differentiation into myelinating OLG. Within the cuprizone-mediated demyelinated CC, OPC express high levels of TNFR2 [35, 39]. Therefore, we next examined whether EHD2-scTNF_R2_ modulates remyelination by enhancing OPC recruitment to the demyelinated areas.

**Fig. 5:**
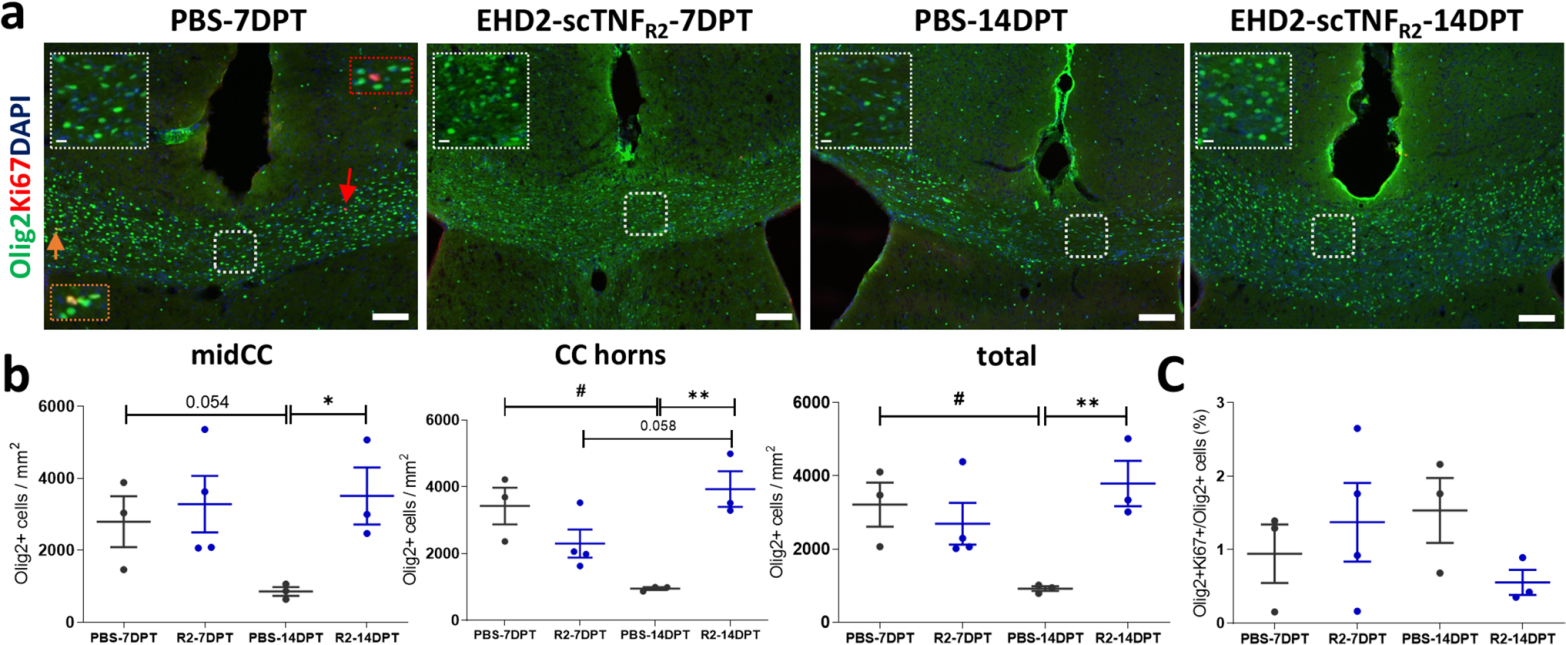
Single EHD2-scTNF_R2_ treatment following cuprizone-induced demyelination results in a higher number of oligodendrocyte lineage cells 14 days after treatment. **a** Representative images of oligodendrocyte lineage marker Olig2 (green) and proliferation marker Ki67 (red) double staining of the corpus callosum (CC) of mice treated either with saline (PBS, grey) or EHD2-scTNF_R2_ (R2, blue) and analyzed 7 or 14 days (DPT) post treatment (DPT). The orange arrow and rectangle point to a Olig2-positive cells that co-express Ki67, the red arrow and rectangle indicate Ki67-positive cells that are negative for Olig2. The percentage of Olig2-positive cells of total cells in the midCC, CC horns*,* and total CC (**b**) and the percentage of Ki67-positive cells of Olig2-positive cells were determined in the total CC (**c**). *n = 3-4 animals/group. An unpaired t-test (c) or Mann-Witney U test (e) were used to compare between two treatments at the same time point (***b***, ****c,*** *p < 0.05, **p < *0.01) or between two time points of the same treatment (***b***, ****c,*** *#p* < *0.05). Scale bars are 100 µm (overview) and 10 µm (insets)*

While not different at 7 DPT, analysis at 14 DPT revealed that the number of Olig2-positive cells, representing OPC and OLG, was more than threefold higher upon EHD2-scTNF_R2_ compared to saline treatment both in the mid CC and CC horns (Fig. 5a, b). In fact, while the number of Olig2-positive cells significantly decreased between 7 and 14 days post saline treatment, oligodendrocyte lineage numbers in EHD2-scTNF_R2_-treated animals were comparable between 7 and 14 DPT, and at 14 DPT significantly higher than in saline-treated animals (Fig. 5b). Co-staining with the proliferation marker Ki67 revealed that the proportion of Olig2-positive cells co-expressing Ki67 was generally low in the CC at 7 and 14 DPT, ranging between 0.5 and 3%, and did not differ between treatments at both time points (Fig. 5c). To assess whether the enhanced numbers of Olig2-positive cells were indeed not a reflection of increased proliferation, we measured the effect of EHD2-scTNF_R2_ on OPC proliferation in vitro. Naïve primary OPC isolated from hu/m TNFR2-ki mice (P1–P3) treated for 24 h one day after plating or for 48 h immediately after plating with EHD2-scTNF_R2_ (Fig. S6a) showed no changes in the number of proliferating Olig2-positive cells (Fig. S7a-c). Hence, these findings demonstrate that the number of oligodendrocyte lineage cells in the CC decreased over time for saline treatment but remained high upon single EHD2-scTNF_R2_ i.c.v. injection without affecting proliferation.

## Discussion

TNFR2 activation in pathological conditions in the CNS elicits homeostatic and regulatory responses that reduce gliosis, neuronal cell death, and demyelination, while enhancing anti-inflammatory and repair processes [4, 5, 40]. Previous researchers employed genetic ablation of TNFR2 [16, 17, 36, 41] or peripheral injection of agonistic molecules in different mouse models relevant to MS [21, 42–44]. However, the consequences of a single pharmacological intervention upon cuprizone-mediated demyelination have not been explored until now. We investigated the impact of a single i.c.v. injection of the TNFR2 agonist EHD2-scTNF_R2_ in hu/m TNFR2-ki mice following cuprizone-induced demyelination on periphery-independent remyelination. In contrast to saline treatment, the percentage of unmyelinated axons in the CC was similar over time upon TNFR2 agonist treatment, indicative of accelerated remyelination. Additionally, single TNFR2 agonist treatment transiently altered the distribution of myelinated axon size towards smaller calibers. At later stages of remyelination, the number of oligodendrocyte lineage cells was higher, and the regenerated myelin around large axons was thinner. Hence, therapeutic CNS activation of TNFR2 by agonists presents a promising approach for modulating remyelination. Further studies should focus on assessing the effects of both single and prolonged TNFR2 agonist stimulation at different stages of the remyelination process and in models where remyelination fails to critically evaluate the therapeutic relevance for MS.

Previous studies established that TNFR2 is essential for remyelination after cuprizone cessation [17], impacting oligodendrocytes by promoting myelin protein synthesis [20], supporting OPC differentiation [16] and enhancing OPC proliferation [17]. We unveiled that a single i.c.v. injection of a TNFR2 agonist following cuprizone-induced demyelination resulted in a lower percentage of unmyelinated axons in the early stages of the remyelination process. In conjunction with a higher myelination compaction score, this suggests accelerated remyelination. Although a potential effect of the TNFR2 agonist or the hu/m TNFR2 knock-in mice on demyelination-related processes cannot be entirely excluded, enhanced remyelination aligns with previous findings. This demonstrates that inhibition of sTNF with BBB-permeable XPro1595 improved myelin compaction and remyelination in both the cuprizone and EAE model, likely via enhanced TNFR2 signaling [11, 16, 18]. In contrast to the effect observed for OPC proliferation with XPro1595 treatment [18], EHD2-scTNF_R2_ treatment did not lead to a significant alteration in the number of proliferating Olig2-positive cells at the examined time points, both in vivo and in vitro. This discrepancy can be explained by several factors. First, TNFR2 modulation by EHD2-scTNF_R2_ occurred at the end of demyelination with the concurrent initial remyelination process, while XPro1595 was administered during demyelination and before the onset of the remyelination process, 2 weeks after cessation of cuprizone feeding. Second, the method of administration (systemic versus i.c.v.) and the duration of modulation (single versus multiple injections) might contribute to the differences observed. Interestingly, a single i.c.v. injection with EHD2-scTNF_R2_ led to a higher number of oligodendrocyte lineage cells in the CC compared to saline treatment at 14 DPT, likely as the number of oligodendrocytes lineage cells significantly drops over time upon saline treatment. In the cuprizone model, the number of OPC initially increases upon cuprizone withdrawal, whereas the number of oligodendrocyte lineage cells decreases upon OPC differentiation [24, 45]. This may indicate an enhanced survival of oligodendrocyte lineage cells upon EHD2-scTNF_R2_ treatment. TNFR2 signaling promotes differentiation of OPC into OLG without affecting their survival in vitro [16]*.* Furthermore, TNFR2 pre-stimulation protected OPC from apoptosis induced by oxidative stress [46]. In the latter in vitro study, TNFR2 was primarily expressed in A2B5-positive OPC with its expression declining after differentiation. To ascertain whether single EHD2-scTNF_R2_ treatment impacts the survival of OPC or mature OLG, a more refined immunohistochemical analysis of the oligodendrocyte lineage, such as the number of OPC, immature, and mature OLG, is warranted.

Other TNFR2-expressing cell types may exert an indirect effect on remyelination. While the activation of TNFR2 in neurons does not affect remyelination upon cuprizone-mediated demyelination [41], TNFR2 stimulation of both microglia and astrocytes played a critical role in diminishing their activation and, concurrently, promoting remyelination [16, 47]. Our data demonstrated that in vitro stimulation of primary microglia with EHD2-scTNF_R2_ increased the expression of anti-inflammatory genes *Arg1* and *Il10*, consistent with a previous study showing that microglia TNFR2 stimulation upregulates anti-inflammatory genes [20]. However, EHD2-scTNF_R2_ treatment did not significantly change the area covered by microglia and astrocytes in the CC. In addition, TNFR2 intervention by i.c.v. injection of an agonist may activate progenitor cells in the adjacent subventricular zone (SVZ), a region contributing to remyelination in the CC [48, 49]. Notably, OPC proliferation in the CC starts around 3 weeks of cuprizone feeding, while the proliferation in the SVZ commences upon cuprizone withdrawal [50]. With this distinction in timing, EHD2-scTNF_R2_ may induce proliferation of progenitor cells in the SVZ [51], potentially recruited to the CC. This may also explain why the number of oligodendrocyte lineage cells remains similar between 7 and 14 DPT in animals treated with EHD2-scTNF_R2_. This hypothesis is substantiated by a previous study showing that stereotactic injection in the lateral ventricle not only affected the neuroinflammatory response around the cannula but also in the cortex and CC, without influencing de- and remyelination process following cuprizone feeding [52]. Hence, the delivery of a TNFR2 agonist in the lateral ventricle may have had a local effect on cells at the injection site, such as differentiation of SVZ progenitor cells into OPC and their subsequent migration to the demyelinated CC.

An unexpected finding is that EHD2-scTNF_R2_ treatment shifted the axon size distribution towards a notably higher frequency of smaller myelinated axons at 7 DPT, without affecting g-ratio and thus myelin biogenesis per se. This finding may imply that EHD2-scTNF_R2_ treatment preferentially supports remyelination of smaller diameter axons early following cuprizone-mediated demyelination. In favor of this explanation, the area covered by myelinated axons at 7 DPT was lower upon EHD2-scTNF_R2_ treatment, while the number of myelinated axons was similar to saline treatment. Alternatively, accelerated covering of axons with compacted myelin may preserve axon shape by halting their swelling, as also noticed for XPro1595 treatment in the EAE model [11]. Intriguingly, in contrast to the saline treatment, upon EHD2-scTNF_R2_ treatment, the g-ratio of large caliber axons between 7 and 14 DPT was not reduced. Given a similar frequency in axon size distribution at 14 DPT, this may point to swelling of myelinated axons after remyelination, without the ability of depositing new myelin membranes to maintain the g-ratio. Previous studies have reported swelling of myelinated axons in cuprizone [53], in EAE [54] and in mutant mice with reduced myelin stability [55]. In our study, the lack of comparison with myelinated axon size distribution in naïve and demyelinated control mice limits the ability to draw definitive conclusions regarding axonal swelling.

Overall, a single treatment with a human TNFR2 agonist directly administered into the brain following cuprizone-induced demyelination in humanized mice modulated the remyelination process at both early and late stages. It appears that the effects measured are milder in comparison with XPro1595 intervention and TNFR2 ablation studies. A possible explanation could be that TNFR2 modulation requires a long-lasting or even pre-stimulation to exert its therapeutic effect on distinct cell types at different stages of the remyelination process. Accordingly, in a previous study, treatment with a TNFR2 agonist yielded stronger effects on motor and sensory disease symptoms when given prophylactically rather than therapeutically in the EAE model [42]. Another possible explanation is the different routes of administration. Little is known about the origin of the beneficial effects of TNFR2-targeting molecules and if their access into the CNS is required. Strong evidence suggests an essential role of regulatory T cell expansion upon TNFR2 stimulation that could have an indirect effect on CNS cells [43, 44, 56, 57], such as type 1 interferon-dependent beneficial activation of microglia [58] and suppression of pathogenic T cells [56]. On the other hand, expression of TNFR2 by glial, neurons, and endothelial cells [6, 11] suggests that local activation in the brain is required. If on one hand the beneficial properties of TNFR2 activation on several pathological hallmarks of MS can be appreciated, substantial effects of this targeting strategy may require modulation of TNF-TNFR2 signaling in both the periphery and CNS. Recent advancements in cell-targeted drug delivery with stimulus-responsive nanocarriers [59] or engineered fusion proteins able to bind to multiple targets [60] can bring further insights into this issue.

## Supplementary Information

Below is the link to the electronic supplementary material.

ESM 1 (DOCX 6.03 MB)

## Data Availability

The STEM datasets generated and analyzed in the current study will be available at full resolution at nanotomy.org. The STEM datasets generated and analyzed in the current study are publicly available at full resolution at nanotomy.org http://www.nanotomy.org. All other data generated during and/or analyzed during the current study are available from the corresponding author upon reasonable request.
