# Supplementary material for: Single intracerebroventricular TNFR2 agonist injection impacts remyelination in the cuprizone model
Source: J Mol Med (Berl). 2025 May 10;103(7):795–807. doi: 10.1007/s00109-025-02549-6 (PMC12287239; doi:10.1007/s00109-025-02549-6)
Supplement: Supplementary file 1 — (DOCX 6.03 MB) [file 109_2025_2549_MOESM1_ESM.docx]

**Supplementary information**

**Single intracerebroventricular TNFR2 agonist injection impacts remyelination in the cuprizone model**

*Valentina Pegoretti, Ate Boerema, Kim Kats, Juan M. Dafauce Garcia, Roman Fischer, Roland E. Kontermann, Klaus Pfizenmaier, Jon D. Laman, Ulrich L.M. Eisel, and Wia Baron^#^*

^#^corresponding author: Wia Baron ([w.baron@umcg.nl](mailto:w.baron@umcg.nl))

**Supplementary Materials and Methods** 2

**Supplementary Table S1** 9

**Supplementary Table 2S**  10

**Supplementary Figure S1** 11

**Supplementary Figure S2** 12

**Supplementary Figure S3** 18

**Supplementary Figure S4** 19

**Supplementary Figure S5** 20

**Supplementary Figure S6** 21

**Supplementary Figure S7** 22

**Supplementary Table S3** 23

**Supplementary Material and Methods**

*Cuprizone model*

Cuprizone-induced demyelination. To induce global demyelination, 8-week-old hu/m TNFR2-ki male mice were fed with a 0.2% cuprizone diet for 5 weeks. The cuprizone diet consisted in a mixture of cuprizone (Sigma-Aldrich, cat. no. C9012), standard chow in powder form and water which was then flattened out, dried and cut in cubes. During cuprizone feeding, mice lost about 10 to 15% body weight which they regained to their baseline within 1 week upon removal of cuprizone from the diet (Fig. S1).

Intracerebroventricular (i.c.v.) injection. At 5 weeks cuprizone feeding, animals were anaesthetized with isoflurane (5% at induction and 1-2% during surgery, in 60% O_2_ enriched air) and immobilized on a Kopf stereotaxic apparatus (Kopf® 900LS) under constant anaesthesia.

To reduce bleeding at the incision site, a subcutaneous injection of lidocaine was performed above the skull. After the incision, a small hole in the skull was performed with a drill followed by an injection with a Hamilton syringe of either saline or EHD2-TNF_R2_ (10 µg in 6.25 µl) dissolved in phosphate-buffered saline (PBS). To determine the dosage, we took into account previous data on peripheral administration, i.c.v. injection volume and molecule aggregation at high concentration. A pump was used to slowly deliver the solution (flow rate: 0.5 µl/min) in the left lateral ventricle (i.c.v. coordinates: -0.05 anteroposterior; -0.1 medial lateral; -0.25 dorsal ventral). After injection, the syringe was slowly removed, and the wound was closed with a suture. When the surgery was completed, the animals were injected with buprenorphine as analgesic (0.05 mg/kg) and placed back in their home cage. Their welfare was monitored for at least 4 h after surgery. Animals returned to standard chow *ad libitum* until sacrifice at 7- or 14-days post treatment (DPT). Surgery and anaesthesia were well tolerated in all mice as seen by their general appearance and body weight increase in the following days (Fig. S1).

Tissue processing. Following an intraperitoneal (IP) injection with a lethal dose of pentobarbital, animals were transcardially perfused, which allowed systemic delivery of a saline solution followed by a fixative solution. For immunohistochemistry, animals were first perfused with heparinized saline solution followed by 4% paraformaldehyde (PFA) in PBS. Brains were removed, post-fixed for 24 h in 4% PFA in PBS, washed several times in PBS and incubated overnight in 30% sucrose solution for cryopreservation. Brains were washed with double distilled water before freezing in liquid nitrogen and storage at -80 °C until use. For electron microscopy, mice were first perfused with Ringer’s solution (135 mM NaCl, 5.4 mM KCl, 1 mM MgCl_2_, 1.8 mM CaCl_2_, 5 mM HEPES, pH 7.4), followed by 4% PFA and 0.1% glutaraldehyde in 0.1 M sodium cacodylate, pH 7.0. Brains were stored at 4 °C in 4% PFA in PBS until further use.

*Immunohistochemistry*

Coronal brain sections (25 µm thick; Bregma between 1.10 mm and 0 mm, Fig. 1a) were cut with a cryostat and stored free-floating in PBS at 4°C. Free-floating sections were pre-incubated for 1 h with 10% normal donkey serum (Jackson Immunoresearch, cat. no. 017-000-121) and 0.5% Triton-X100 in PBS (blocking solution) to avoid unspecific binding and increase permeability, respectively. Then, sections were incubated with appropriate primary antibodies (Supplementary table 1) diluted in blocking solution at 4°C either overnight (GFAP, Olig2) or for 72 h (IBA1) or at 37°C for 1 h (Ki67). To increase accessibility of the anti-MBP antibody for its epitope, a pre-incubation of 30 min in ethanol/acetic acid solution was required. Three washing steps preceded secondary antibody incubation (Supplementary table 1) for 2 h at room temperature (RT). For visualization with DAB, sections were incubated with avidin-biotin complex (ABC, 1:500; Vector Laboratories, cat. no. PK-4000) for 1 h at RT, followed by three washing steps and incubation with 3,3′-diaminobenzidine-tetrahydrochloride (DAB, 0.7 mg/ml; Sigma-Aldrich, cat. no. D4293) to generate a brown precipitate. For visualization of nuclei with fluorescence, sections were incubated with 0.1 µg/ml DAPI (Thermo Fisher Scientific, cat. no. D1306) for 3 min. Each washing step was performed with 0.5% Triton-X100 in PBS for 5 min. For both fluorescent and DAB visualization, a Leica DMI6000 B fluorescence microscope was used to capture mosaic images of whole brain sections at a 10x magnification. Overall signal coverage of MBP, IBA1 or GFAP staining in the CC were measured using ImageJ (Fig. S2a) [1]. Signal coverage of IBA1 and GFAP staining were also measured in the whole section. Signal coverage reflects only the surface area of staining but not signal intensity or cellular localization. Olig2-positive cells in three areas of the CC (two horns and mid CC) were manually counted in Image J. Similarly, Olig2- and Ki67-positive cells were manually counted in the whole CC while IBA1- and GFAP-positive cells in two areas of the cingulate cortex (Fig. 1a).

*Black gold II myelin stain*

Coronal brain sections were mounted directly onto glass object slides after sectioning and stained with Black Gold II (BGII) myelin staining kit (3 mg/ml; Sigma-Aldrich, cat. no. AG105), following manufacturer’s instructions. BGII staining was imaged using Olympus BH2 microscope (Leica QWin Software) and optical density in the CC of three sections per animal was measured using ImageJ software. Grey values were converted into relative optical density (OD) based on the camera’s properties.

*Scanning transmission electron microscopy* (STEM)

Image acquisition. Samples were prepared for nanotomy essentially the same as described for STEM analysis of human pancreas [2] and brain samples [3]. After a first post-fixation with 2% PFA and 2% GA in 0.1 M sodium cacodylate pH 7, a small tissue block coinciding with the CC horn was separated, rinsed three times with 0.1 M cacodylate buffer and post-fixed in 1% osmium and 1% K_4_ [Fe(CN)_6_] solution for 2 h. The samples were dehydrated and embedded in epoxy [2]. 80 nm thick sections were stained with uranyl acetate (Merck, cat. no. 8473) and imaged with a scanning transmission electron microscope (STEM; Zeiss® Supra 55). For the high-resolution STEM, samples were cut at 50 nm thickness, stained with Neodymium acetate (Aldrich PCode: 1002642860) and imaged with a TalosF200i at 200Kv, spot size 6, gun lens 4 and C2 aperture 70 µm. Full resolution images are available at <https://www.nanotomy.org>.

Manual analysis. To analyze the cross-sectional ultrastructural features or individual (un)myelinated axons, several randomly selected zoomed-in pictures (pixel size 7.4nm) per animal were taken for precise measurements in ImageJ software (<https://imagej.net/ij/index.html>). Selection was performed by placing a grid over the whole scanned image and random rectangles (approximately 10 µm x 4 µm each) were picked for analysis. Myelinated axons were then counted. Further, the inner area and the myelin thickness of the axon were drawn and measured. The axon diameter was back calculated as 2*√(area/π) and the fibre diameter was considered as the sum of the axon diameter and twice the myelin thickness). The g-ratio index was calculated as the ratio between the axon and the fibre diameter [4]. For the analysis of mitochondria, area and number of mitochondria per myelinated axon were analyzed and presented as total axonal mitochondrial area, percentage of the axonal area (mitochondrial content) and the percentage of myelination axons with mitochondria. For the analysis of high-resolution STEM images, axons and radial component were manually counted. For manual analysis, a minimum of 100 axons in about 6 to 8 randomly picked rectangles were measured per animal in a blinded fashion. Axons that were too oval, longitudinally cut, and/or with uncompacted myelin lamellae on all sides were excluded from the analysis.

Semi-automated analysis. Parallel to manual analysis, we developed a semi-automated method based on automatic segmentation of the images using a seeded region growing algorithm (SRG; MATLAB code in Fig. S2b) [5]. The SRG algorithm selected a region based on the intensity of the pixels. In this algorithm, multiple seeds were selected per image based on the grayscale histogram. The second highest peak of the histogram corresponded to pixels from the myelin sheath and thus, every pixel with the intensity of the peak was used as a seed. The difference in intensity to define when the myelin sheath ends was calculated as follows:

**𝐷 = 𝑇 − 𝐼_𝑠_ + 𝐶**

where D is the allowed difference, T is the optimal threshold according to Otsu’s method, I_s_ is the intensity of the seeds and C is a small correction factor. Once the mask was complete, all particles with less than 1.000 pixels were filtered out and artefacts (areas erroneously labelled as myelin, axons on the edges of the image and longitudinally cut axons) were manually removed in Image J. Finally, the intensity of myelin was set to 128 and the area of axons filled at an intensity of 255. For each image, total pixels and pixels recognized as myelin or axon were then counted by a second algorithm (Fig. S2c) and converted into area. With the SRG algorithm, all myelin and axon areas in one image were put together into a single fibre from which the aggregated g-ratio of all myelinated axons was calculated. For each sample, 15 to 30 square areas were semi-randomly distributed and picked for analysis (approximately 300 to 2.000 cross-sectional axons/sample). Notably, this semi-automated method determines the aggregated g-ratio by dividing axonal area by fibre area.

*Primary cell cultures*

Mixed glia cultures. Cortices of newborn hu/m TNFR2-ki animals (P1-P3) were digested with MEM supplemented with papain (30 U/ml; Sigma-Aldrich, cat. no. P3125), 240 µg/ml L-cysteine (Sigma-Aldrich, cat. no. C7477) and 40 µg/ml DNase (Roche, cat. no. 10104159001) at 37 °C for 50 min. Tissue digestion was stopped with two times incubation with trypsin inhibitor (1 mg/ml) and bovine serum albumin (BSA, 50 µg/ml; Sigma-Aldrich, cat. no. A4919) in L15 medium for 3 min at RT. Cells were then washed with medium (MEM with 1% penicillin/streptomycin, P/S) and resuspended in DMEM with 1% P/S, 1% L-glutamine and non-heat inactivated 10% fetal bovine serum (FBS; Capricorn Scientific, cat. no. FBS-12A) before plating in poly-L-lysine (PLL, 5 μg/ml; Sigma-Aldrich, cat. no. P2636)-coated T75 flasks. After two weeks, enriched microglia and OPC cultures were obtained by a sequential shake off procedure.

Microglia. Microglia were first dissociated from the confluent astrocyte layer by a 1 h shake off using an orbital shaker (Innova 4000, 150 rpm, 37 °C). Cells were plated in 6-well plates at a concentration of 10^6^ cells/well in DMEM supplemented with 10% FBS. Microglia at a purity over 95% (IBA1-positive cells) were treated with EHD2-scTNF_R2_ (100 ng/ml) or PBS for 24 h from the day of plating.

OPC. OPC were detached from the astrocyte monolayer by an overnight shake off (240 rpm, 37 °C). To enrich for OPC, medium with floating cells was transferred to a non-tissue culture petri dish and incubated for 15 min at 37°C to allow microglia and astrocytes to adhere. The cells were plated on 13 mm PLL-coated coverslips in 24 well plates at a concentration of 40,000 cells/well in SATO medium [6,7]. 1 h after plating, platelet-derived growth factor-AA (PDGF-AA, 10 ng/ml; Peprotech, cat. no. 100–13) and fibroblast growth factor-2 (FGF2, 10 ng/ml; Peprotech, cat. no. 100-18) were added to stimulate OPC proliferation. OPC at around 70% purity (Olig2-positive cells) were treated with PBS or EHD2-scTNF_R2_ (100 ng/ml) at time of plating (0 days *in vitro* - DIV) for 48 h or at 1 DIV for 24 h.

*Immunocytochemistry*

Primary OPC were fixed with 4% PFA in PBS for 15 min at RT. Cells were then washed three times with PBS and incubated with 4% BSA in PBS for 30 min to block non-specific antibody binding sites followed by incubation for 1 h with primary antibodies (see Supplementary table 1) diluted in blocking solution. Following three washing steps with PBS, cells were incubated with appropriate FITC-/TRITC-conjugated secondary antibodies (Jackson Immunoresearch, 1:50) and 1 µg/ml of DAPI for 30 min. Cells were washed three times with PBS, mounted (Dako mounting medium) and analyzed in a blinded fashion using an immunofluorescence microscope (Leica DMI 6000 B). In each independent cell culture experiment, three images per treatment condition from two technical replicates were analyzed by manually counting Olig2- and/or Ki67-positive nuclei (around 300 DAPI nuclei per coverslip, two independent cell culture experiments).

*RNA extraction and quantitative real-time PCR*

Primary microglia were harvested as a pellet, homogenized and total RNA was extracted using Monarch® Total RNA Miniprep Kit (New England Biolabs). Total RNA was measured using NanoDrop spectrophotometer (IMPLEN NanoPhot_16). RNA was reverse transcribed in 100 ng/µl cDNA using High-Capacity cDNA Reverse Transcription Kit (Thermo Fisher Scientific, cat. no. 4368814). Transcript abundance was determined by using PowerUp™ SYBR™ Green Master Mix (Thermo Fisher Scientific, cat. no. A25742) with the primer pairs specific for genes of interest (Supplementary Table 2) and relative changes in gene expression were calculated with the ΔΔCt method where the house-keeping gene *Rplp0* was used.

**References**

[1] Schneider CA, Rasband WS, Eliceiri KW (2012) NIH Image to ImageJ: 25 years of image analysis. Nat Methods 9:671–675. https://doi.org/10.1038/nmeth.2089

[2] de Boer P, Pirozzi NM, Wolters AHG, Kuipers J, Kusmartseva I, Atkinson MA, Campbell-Thompson M, Giepmans BNG (2020) Large-scale electron microscopy database for human type 1 diabetes. Nat Commun 11:2475. https://doi.org/10.1038/s41467-020-16287-5

[3] Oost W, Huitema AJ, Kats K, Giepmans BNG, Kooistra SM, Eggen BJL, Baron W (2023) Pathological ultrastructural alterations of myelinated axons in normal appearing white matter in progressive multiple sclerosis. Acta Neuropathol Commun 11:100. https://doi.org/10.1186/s40478-023-01598-7

[4] Moore AC, Mark TE, Hogan AK, Topczewski J, Leclair EE (2012) Peripheral axons of the adult zebrafish maxillary barbel extensively remyelinate during sensory appendage regeneration. J Comp Neurol 520:4184–4203. https://doi.org/10.1002/cne.23147

5] Zhao X, Pan Z, Wu J, Zhou G, Zeng Y (2010) Automatic identification and morphometry of optic nerve fibers in electron microscopy images. Comput Med Imaging Graph 34:179–184. https://doi.org/10.1016/j.compmedimag.2009.08.009

[6] Lentferink DH, Jongsma JM, Werkman I, Baron W (2018) Grey matter OPCs are less mature and less sensitive to IFNγ than white matter OPCs: Consequences for remyelination, Sci Rep 8:2113. https://doi.org/10.1038/s41598-018-19934-6

[7] Maier O, van der Heide T, van Dam AM, Baron W, de Vries H, Hoekstra D (2005) Alteration of the extracellular matrix interferes with raft association of neurofascin in oligodendrocytes. Potential significance for multiple sclerosis? Mol Cell Neurosci 28:390–401. https://doi.org/10.1016/j.mcn.2004.09.012

**Table S1** Primary and secondary antibodies for immunochemistry

| **antibody** | **type** | **host** | **cat. no.** | **company** | **dilution^a^** |
| --- | --- | --- | --- | --- | --- |
| MBP | primary | rat | MAB386 | Millipore | 1:100 |
| Olig2 | primary | mouse | MABN50 | Sigma-Aldrich | 1:1,000 (IHC) / 1:100 (ICC) |
| Ki67 | primary | rat | 14-5698-82 | Thermo Fisher Scientific | 1:300 (IHC) / 1:1,000 (ICC) |
| IBA1 | primary | rabbit | 019-19741 | Wako Chemicals | 1:2,500 (IHC) / 1:250 (ICC) |
| GFAP | primary | mouse | G3893 | Sigma-Aldrich | 1:10,000 (IHC) / 1:500 (ICC) |
| anti-mouse IgG Alexa 488 | secondary | donkey | A21202 | Thermo Fisher Scientific | 1:600 |
| anti-rat IgG FITC | secondary | donkey | A18740 | Thermo Fisher Scientific | 1:100 |
| anti-rat IgG Alexa 555 | secondary | goat | A21434 | Thermo Fisher Scientific | 1:600 |
| anti-rabbit IgG biotin-conjugated | secondary | goat | 111-065-003 | Jackson ImmunoResearch | 1:500 |
| anti-mouse IgG biotin-conjugated | secondary | goat | 115-065-003 | Jackson ImmunoResearch | 1:500 |

^a^ IHC – immunohistochemistry; ICC - immunocytochemistry

**Table S2** Primer sequences for qPCR

| **gene**  (mus musculus) | **NCBI reference sequence** | **product length (bp)** | **sequence** |
| --- | --- | --- | --- |
| tumor necrosis factor *(Tnf)* | NM_013693.3 | 132 | F: 5’ CCCCAAAGGGATGAGAAGTT 3’ |
|  |  |  | R: 3’ CACTTGGTGGTTTGCTACGA 5’ |
| interleukin-1β (*Il1b)* | NM_008361.4 | 175 | F: 5’ GGGCCTCAAAGGAAAGAATC 3’ |
|  |  |  | R: 3’ GGGGAACTCTGCAGACTCAA 5’ |
| nitric oxide synthase 2 (*Nos2*) | NM_001313922.1 | 105 | F: 5’ GGAAGAAATGCAGGAGATGG 3’ |
|  |  |  | R: 3’ TGCAGGATGTCCTGAACGTA 5’ |
| arginase-1 (*Arg1*) | NM_007482.3 | 155 | F: 5’ GAACACGGCAGTGGCTTTAAC 3’ |
|  |  |  | R: 3’ TGCTTAGCTCTGTCTGCTTTGC 5’ |
| interleukin-10 (*Il10*) | NM_010548.2 | 105 | F: 5’ GCTCTTACTGACTGGCATGAG 3’ |
|  |  |  | R: 3’ CGCAGCTCTAGGAGCATGTG 5’ |
| ribosomal protein, large, P0 (*Rplp0)* | NM_007475.5 | 121 | F: 5’ TCACTGTGCCAGCTCAGAAC 3’ |
|  |  |  | R: 3’ ATCAGCTGCACATCACTCAGA 5’ |

**
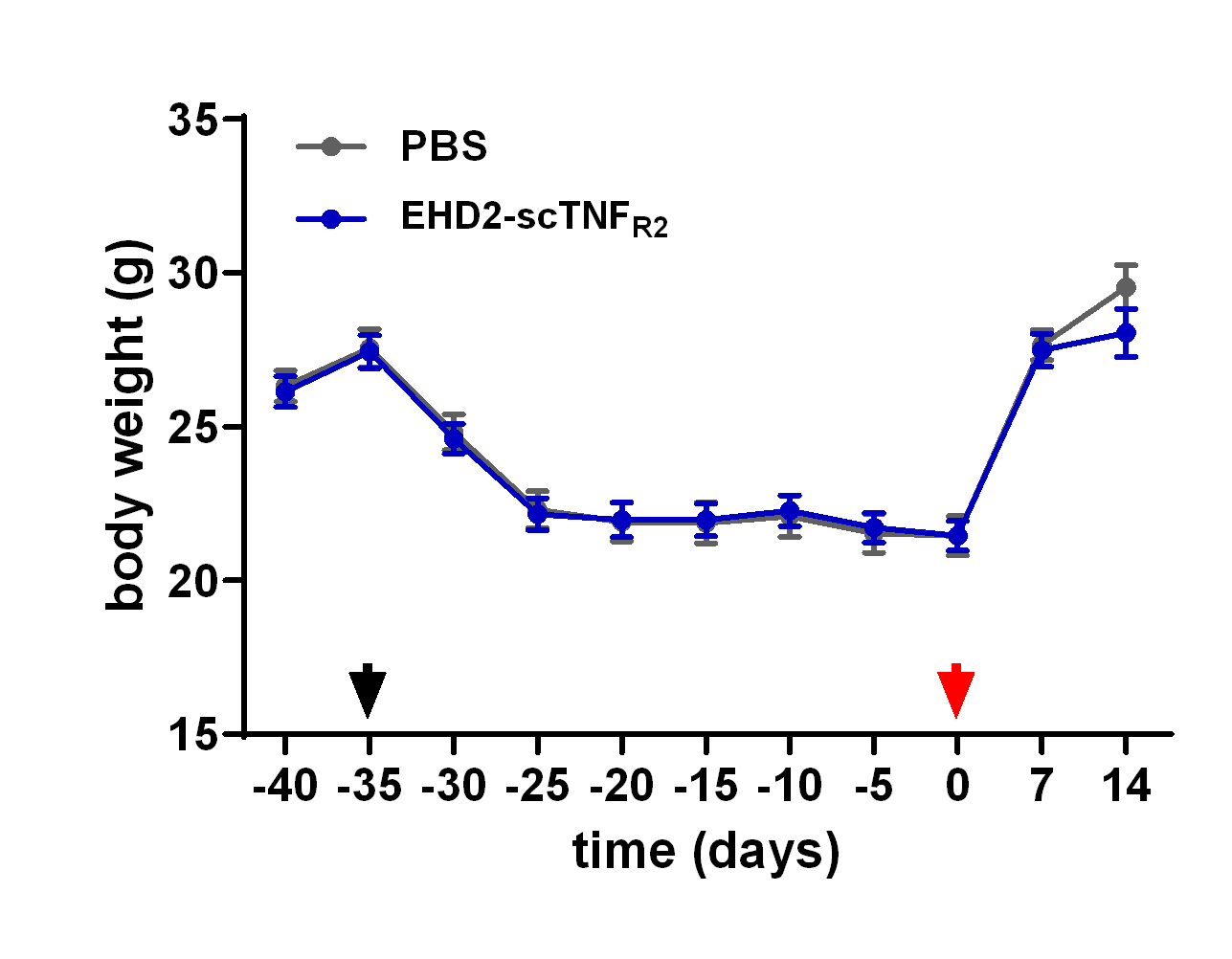
**

**Fig. S1** Bodyweight changes during cuprizone feeding and following treatment. Hu/m TNFR2-ki mice were weighed during cuprizone feeding and following single intracerebroventricular (i.c.v.) injection with saline (PBS, grey) or EHD2-scTNF_R2_ (blue) every 5 to 7 days. The average body weight (g, *n = 6-8 animals/group*) is plotted. The black arrow indicates the start of cuprizone feeding and the red arrow marks the end of cuprizone feeding and day of i.c.v. injection. Data are presented as mean ± SEM*. A two-way ANOVA with Sidak’s multiple comparison test was conducted to compare between treatments at each time point*

**Fig. S2** ImageJ macros. ImageJ macros used to measure signal coverage of MBP, IBA1 or GFAP in microscopy images (**a**), MATLAB codes for detecting myelin (**b**) or measuring the SRG mask (**c**) in STEM images.

(**a**)

run("Subtract Background...", "rolling=50 light");

run("Add Selection...");

setOption("BlackBackground", false);

run("Convert to Mask");

run("To ROI Manager");

roiManager("Select", 0);

roiManager("Measure");

(**b**)

%%

clear variables

close all

%% Prealocation of memory

tic %First part of timing the script

%Making it easier to use for all of our sections

path=uigetdir('Choose the folder containing the images: '); %NOTE THAT FILES ARE NOT VISIBLE

%Chosen directory must include the following sub-directories:

% SRG_Mask, SRG_Mask+Image, Unfiltered

prefix=input('Input the name of the image set (excluding enumeration of images): ','s'); %All images in the same set should follow the naming scheme "(name)1,(name)2, etc."

noi=input('Input the number of images in the set: '); %Prealocation of memory to make it faster

goodi=input('Input the number of the image that will be used for myelin thresholding: '); %Not all images are apt for thresholding

contrastq=input('Adjust tolerance (0-255): '); %For low contrast images a incresing or decreasing tolerance makes for a better result

contraste=(contrastq/255); %Change the scale from 1-255 to 0-1. 7/255 = 0.0275

imagenD{noi}=0;

imagenI{noi}=0;

FullMyelin{noi}=0;

alto(noi)=0;

ancho(noi)=0;

FilteredMyelin{noi}=0;

%% We import every image and copy them into double

for n=1:noi

imagenI{n}= imread(sprintf('%s/%s%01d.png',path,prefix,n));

imagenD{n}= im2double(imagenI{n});

end

%% We define the myelin pixel intensity and the threshold between myelin and background

[counts,binLocations] = imhist(imagenI{goodi}); %We get the grey level histogram

figure('Name','Histogram plot of reference image','NumberTitle','off')

pixeldistribution=plot(binLocations,counts); %We plot the raw histogram (linear plot)

extrasuave=smooth(smooth(counts)); %Double smoothing of the histogram, for a better result

smoothpixels=plot(binLocations, extrasuave,'r'); %We plot the smoothed histogram

%The biggest peak correspond to cytoplasm, the second biggest corresponds

%with myelin. If the second peak is not well defined the program won't

%properly detect it, and you will have to manually input it

Thresh1=graythresh(imagenI{goodi}); %We calculate the threshold according to the Otsu method

%Thresh1=0.3921; %Manual threshold input

Thresh255=Thresh1*255; %Put it in a scale from 0 to 255

[countvalue, pxvalue]= findpeaks(extrasuave); %We get the x and y values of the peaks

[sortedValues, sortIndexes] = sort(countvalue, 'descend'); %We sort the peaks according to height

sortedpxvalues=pxvalue(sortIndexes,:); %We sort the values in x(intensity) based on the values of y(frequency/number of

pixels)

seedintensity255=sortedpxvalues(2); %We get the intensity of the pixels in the second highest peak (which corresponds to

myelin)

%seedintensity255=input('Input seed intensity (0-255): '); %Manual seed intensity input, for sections where the peaks are

weird

seedintensity1=seedintensity255/255;

%We define the maximum difference between the intensity of the pixel and its neighbor,

%with a slight adjustment in the case of low contrast images that makes the allowed difference smaller

alloweddifference=Thresh1+contraste-seedintensity1;

%% We apply the region growing algorithm to each image

for n=1:noi

%% We find the x and y coordinates for the seeds

[seedx, seedy]=find(imagenI{n}==seedintensity255); %We create 2 vectors, with the positions of all the pixels with the

desired value

[alto(n), ancho(n)]=size(imagenD{n}); %We calculate the size of the image for memory preallocation

JFull=zeros(alto(n),ancho(n)); % Output

I=imagenD{n}; %We put the image we're working on into the variable I

for a=1:length(seedx)

x=seedx(a);

y=seedy(a);

if JFull(x,y)==0 %Make sure the seed isn't part of the image yet

%% The big part: we perform the algorithm from each seed, and add the results all together

J = zeros(size(I)); % Output

Isizes = size(I); % Dimensions of input image

reg_mean = I((x(1)),(y(1))); % The mean of the segmented region

reg_size = 1; % Number of pixels in region

% Free memory to store neighbours of the (segmented) region

neg_free = 10000; neg_pos=0;

neg_list = zeros(neg_free,3);

pixdist=0; % Distance of the region newest pixel to the regio mean

% Neighbor locations (footprint)

neigb=[-1 0; 1 0; 0 -1;0 1];

% Start regiogrowing until distance between region and posible new pixels become

% higher than a certain treshold

while(pixdist<alloweddifference&&reg_size<numel(I))

% Add new neighbors pixels

for j=1:4

% Calculate the neighbour coordinate

xn = x +neigb(j,1); yn = y +neigb(j,2);

% Check if neighbour is inside or outside the image

ins=(xn>=1)&&(yn>=1)&&(xn<=Isizes(1))&&(yn<=Isizes(2));

%Check if the intensity of the point is closer to the seed than the

%seed to the threshold,

if ins; difference=(abs(I(xn,yn)-seedintensity1)<=alloweddifference & I(xn,yn)<=0.99);end

% Add neighbor if inside and not already part of the segmented area, and if the previous condition is fulfilled

if(ins&&difference&&(J(xn,yn)==0))

neg_pos = neg_pos+1;

neg_list(neg_pos,:) = [xn yn I(xn,yn)];

J(xn,yn)=1;

end

end

if neg_pos==0 %If no neighbors can be added, this prevents the code for looping infinitely

break;

end

% Add a new block of free memory

if(neg_pos+10>neg_free), neg_free=neg_free+10000; neg_list((neg_pos+1):neg_free,:)=0; end

% Add pixel with intensity nearest to the mean of the region, to the region

dist = abs(neg_list(1:neg_pos,3)-reg_mean);

[pixdist, index] = min(dist);

J(x,y)=2; reg_size=reg_size+1;

% Calculate the new mean of the region

reg_mean= (reg_mean*reg_size + neg_list(index,3))/(reg_size+1);

% Save the x and y coordinates of the pixel (for the neighbour add proccess)

x = neg_list(index,1); y = neg_list(index,2);

% Remove the pixel from the neighbour (check) list

neg_list(index,:)=neg_list(neg_pos,:); neg_pos=neg_pos-1;

end

% Return the segmented area as logical matrix

J=J>1;

JFull=JFull+J;

end

end

%% To save the results

imwrite(JFull, sprintf('%s/Unfiltered/Region growing mask only for %s%01d.png',path,prefix,n)) %This mask may include

many small artifacts, but could still be usefull when myelin is patchy

%% Filtering

FilteredMyelin=bwareaopen(JFull,1000); %We filter out regions of under 1000 pixels to remove small artifacts. This

number is abritrary and depends on pixel size and myelin integrity

imwrite(imagenD{n}+FilteredMyelin, sprintf('%s/SRG_Mask+Image/RG mask with image for %s%01d.png',path,prefix,n))

%We save the mask overlapping the corresponding image

imwrite(FilteredMyelin, sprintf('%s/SRG_Mask/RG mask for %s%01d.png',path,prefix,n)) %We save the raw mask

end

%%

Duration=toc; %Second part of timing the script

%We need to clean the images before the next step

(**c**)

clear variables

close all

%% AFTER CLEANUP

path=uigetdir('Choose the folder containing the images: '); %NOTE THAT FILES ARE NOT VISIBLE

%Chosen directory must include the following directories:

% SRG_Mask_Cleaned(where the cleaned images are stored), SRG_Mask_Cleaned+Image

prefix=input('Input the name of the image set (excluding enumeration of images): ','s'); %Cleaned masks in should be named

like the original masks with the suffix "_Cleaned" added

noi=input('Input the number of images in the set: '); %Prealocation of memory to make it faster

pxlsize=input('Input pixel size: '); %Pixel area will be calculated from pixel size

alto(noi)=0;

ancho(noi)=0;

AxonPixels(noi)=0;

AxonArea_nm(noi)=0;

MyelinPixels(noi)=0;

MyelinArea_nm(noi)=0;

TotalPixels(noi)=0;

TotalArea_nm(noi)=0;

for n=1:noi

%% Importing

imagenI= imread(sprintf('%s/%s%01d.png',path,prefix,n));

Masks= imread(sprintf('%s/SRG_Mask_Cleaned/RG mask for %s%01d_Cleaned.tif',path,prefix,n));

MasksI= uint8((Masks==128).*255);

%We save the cleaned mask overlapping the image for future reference

imwrite(imagenI+MasksI, sprintf('%s/SRG_Mask_Cleaned+Image/RG mask with image for

%s%01d_Cleaned.tif',path,prefix,n))

%% Measuring

MyelinPixels(n)=sum(Masks(:)==128); %Values of 128 are myelin

AxonPixels(n)=sum(Masks(:)==255); %Values of 255 are axons

[alto(n), ancho(n)]=size(Masks);

TotalPixels(n)=alto(n).*ancho(n);

MyelinArea_nm(n)=MyelinPixels(n).*(pxlsize^2); %We convert to area by multiplying pixel numbers by pixel area (pixel

size squared)

TotalArea_nm(n)=TotalPixels(n).*(pxlsize^2);

AxonArea_nm(n)=AxonPixels(n).*(pxlsize^2);

end

save(sprintf('%s/%s_Data.txt',path,prefix),'MyelinArea_nm','TotalArea_nm', 'AxonArea_nm', '-ascii') %The collected data is saved in text form. Note that each line corresponds to a different stat, and each column to a different individual image

*
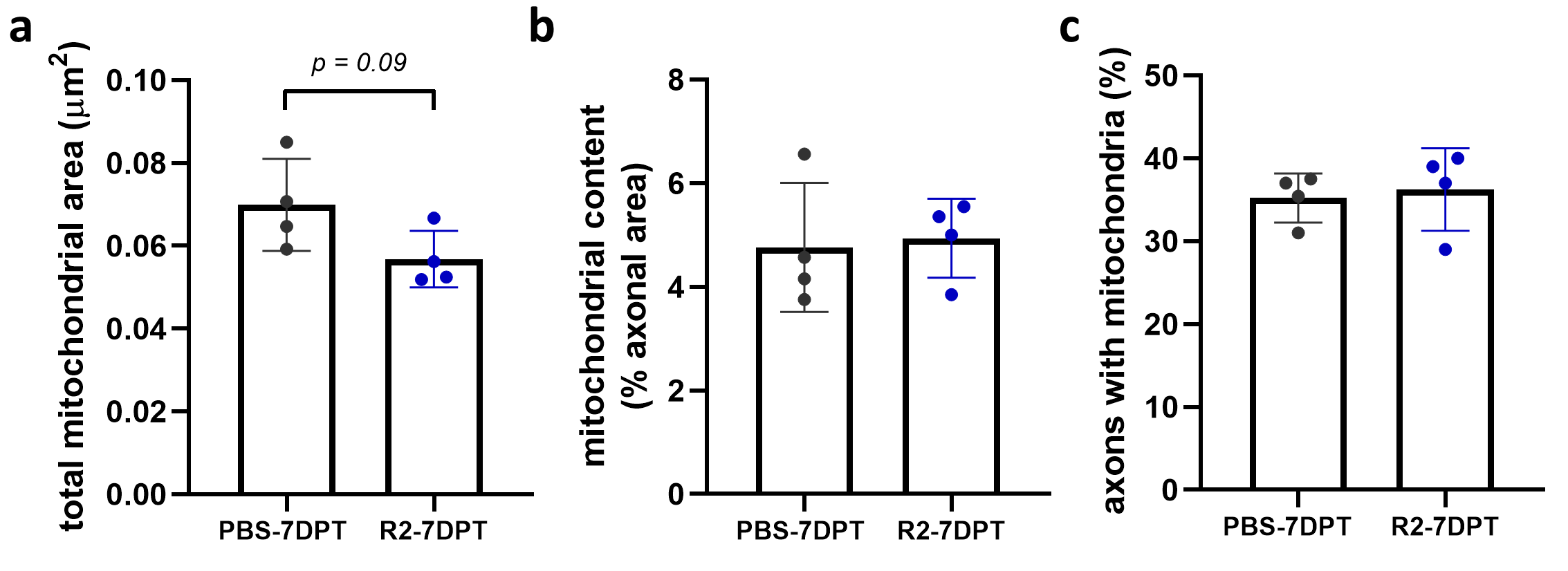
*

**Fig. S3** Single EHD2-scTNF_R2_ treatment following cuprizone-induced demyelination tends to reduce cross-sectional mitochondrial area in myelinated axons at 7 days post treatment. STEM image analysis of the corpus callosum of mice treated either with saline (PBS, grey) or EHD2-scTNF_R2_ (R2, blue) and 7 days post treatment (DPT). For each animal, total cross-sectional axonal mitochondrial area (**a**), mitochondrial content expressed as percentage of the axonal area (**b**) and the percentage of myelination axons with mitochondria (**c**) were measured. At least 100 axons per animal were manually measured. *n = 4 animals/group. An unpaired t-test was conducted to compare between the two treatments.*

**
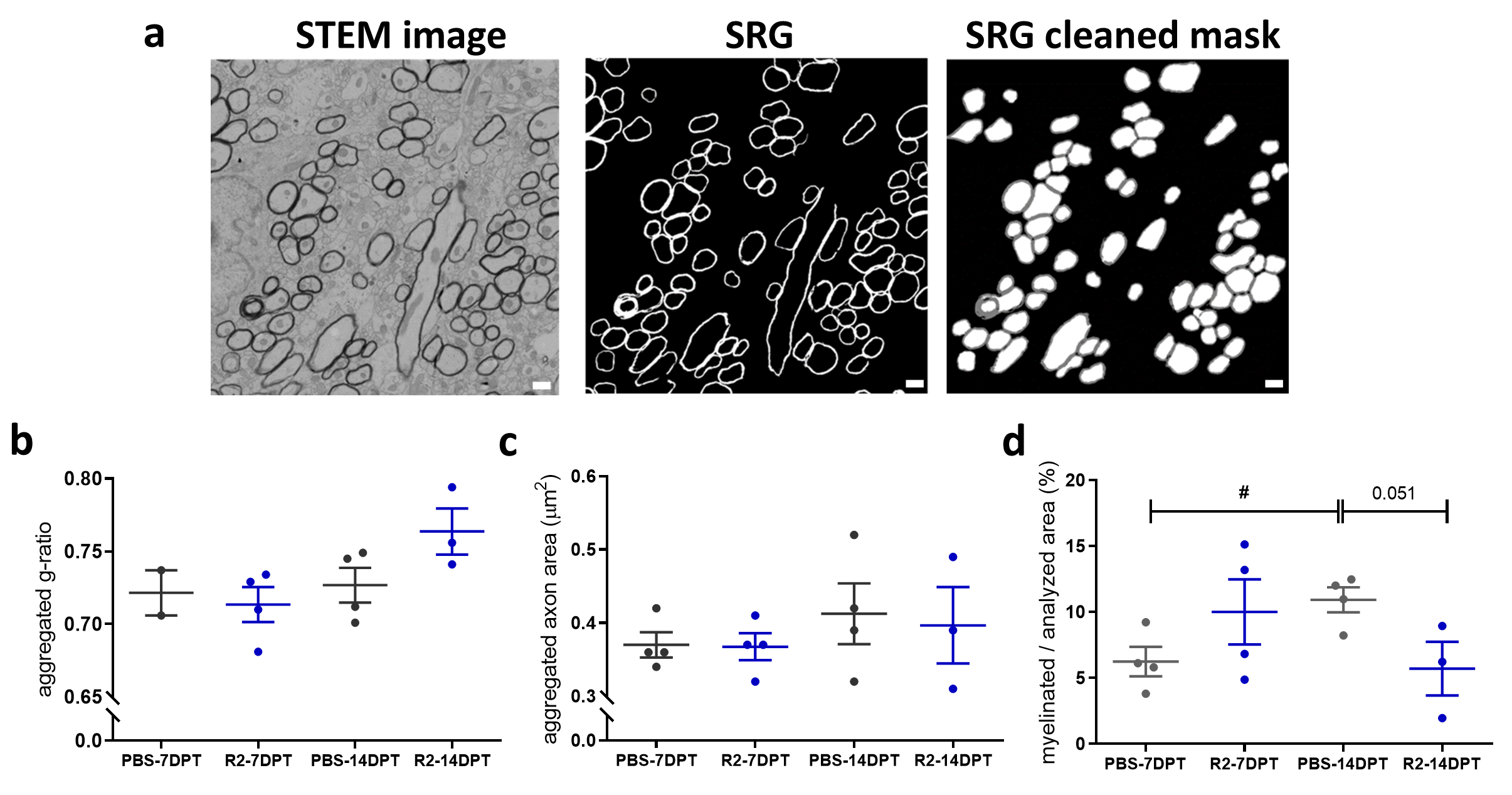
**

**Fig. S4** Seeded region growing (SRG) algorithm quantification of aggregated g-ratio, axon diameter and the percentage of myelinated area. **a** Representative scanning transmission electron microscopy (STEM) input and output images of the SRG algorithm before and after irrelevant areas are excluded. The aggregated g-ratio (**b**), aggregated axon area (**c**) and percentage of myelinated area (**d**) in hu/m TNFR2-ki mice after single intracerebroventricular (i.c.v.) injection with saline (PBS, grey) or EHD2-scTNF_R2_ (R2, blue) following cuprizone-induced demyelination analyzed at 7- or 14-days post treatment (DPT) are shown. *n = 3-4 animals/group. An unpaired t-test (****b****,* ***c****) or Mann-Whitney U (****d****) test was used to compare between two treatments at the same time point (****b****-****d****, not significant) or two time points of the same treatment (****b-d****, #p < 0.05). Scale bars are 1 μm*

*
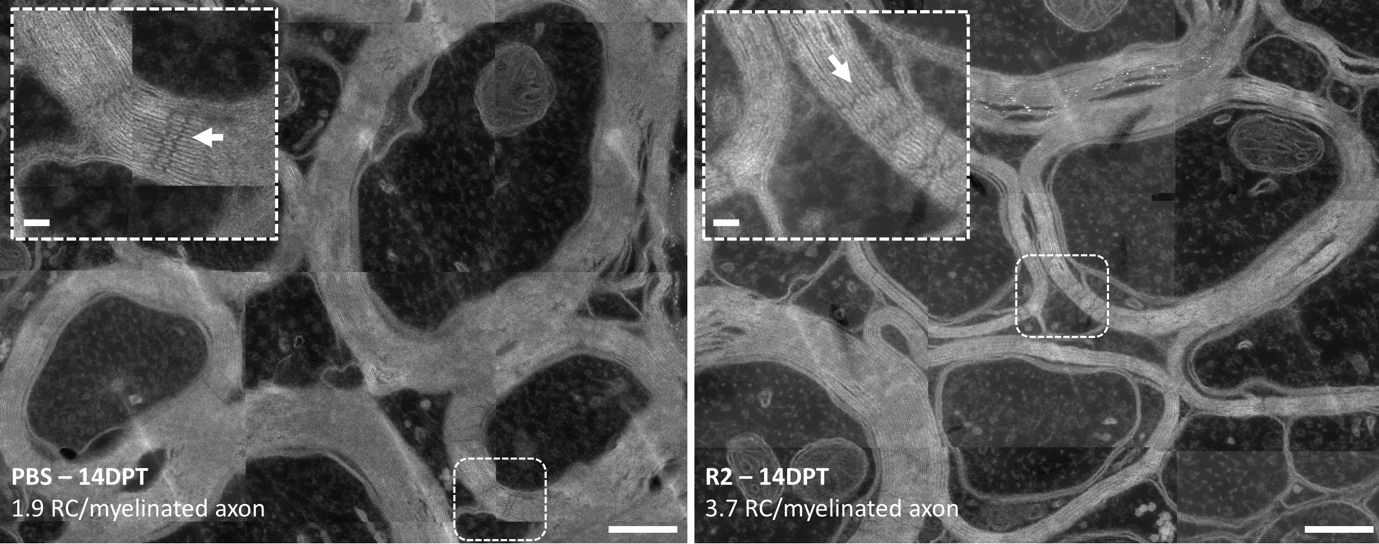
*

**Fig. S5** Single EHD2-scTNF_R2_ treatment following cuprizone-induced demyelination results in more radial component and fewer myelin membrane layers at 14 days post treatment. Representative areas of a high-resolution STEM image of the corpus callosum of a mouse treated with saline (PBS, left) or EHD2-scTNF_R2_ (R2, right) at 14 days post treatment (14 DPT). Data is available at full resolution and scale at nanotomy.org. The number of radial component (RC) in each scan was manually counted (PBS: 1.9 RC / myelinated axon [83 RC in 43 axons; 72.1% of axons with RC]; EHD2-scTNF_R2_: 3.7 RC / myelinated axon [136 RC in 37 axons; 91.9% of axons with RC]). *Scale bars are 0.5 µm (overview) and 10 nm (inset)*

**
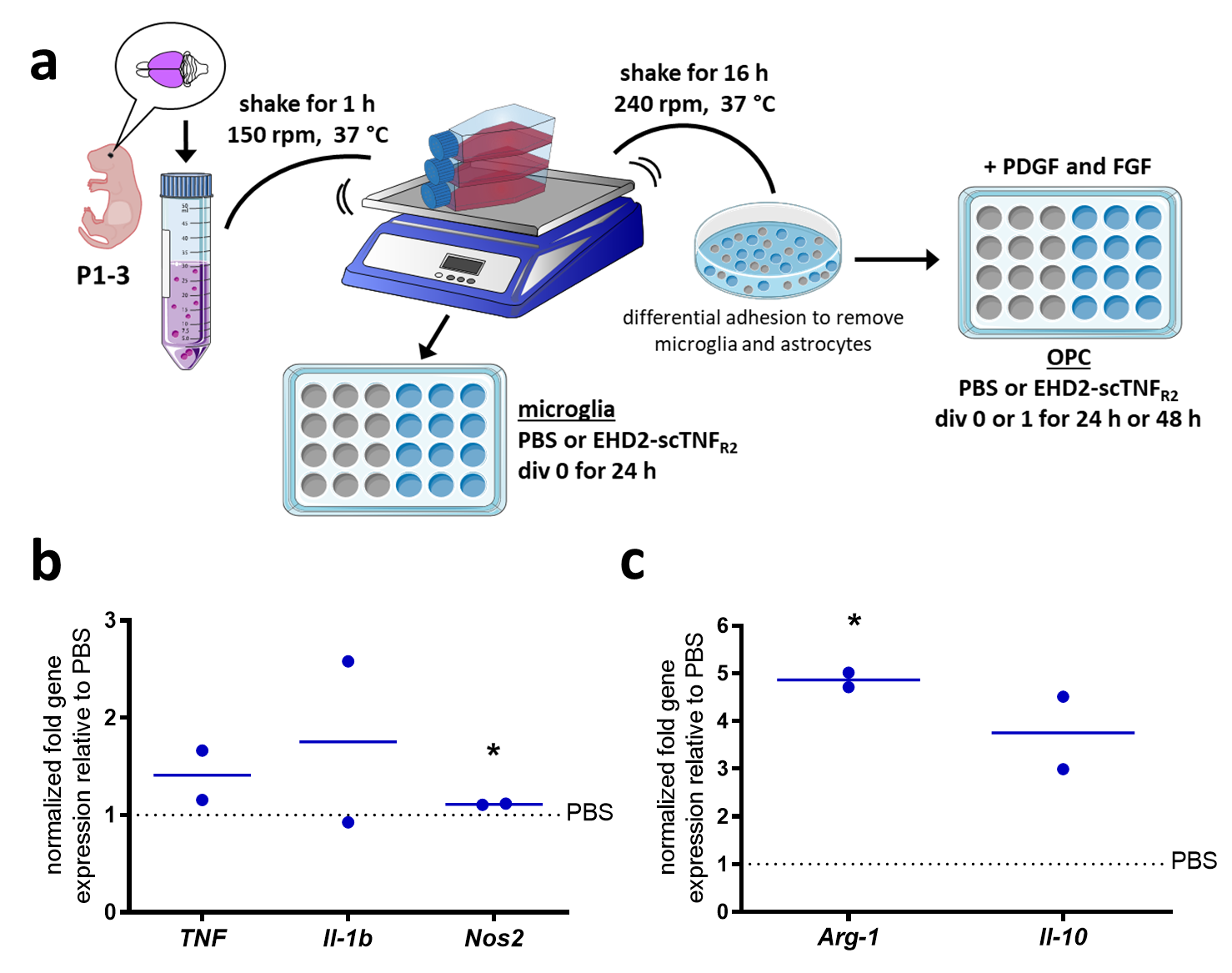
**

**Fig. S6** EHD2-scTNF_R2_ treatment increases mRNA expression of anti-inflammatory genes in primary microglia. **a** Schematic representation of the in vitro experimental set up. (**b,c**) Primary microglia isolated from hu/m TNFR2-ki mice were treated with PBS or EHD2-scTNF_R2_ and after 24 h subjected to qPCR. (**b**) mRNA expression of pro-inflammatory cytokines *TNF*, *Il1β*, and *Nos2*. (**c**) mRNA expression of anti-inflammatory cytokines *Arg1* and *Il10*. Data is presented as mean value of two independent cell culture experiments relative to PBS (horizontal line, set at 1 in each independent cell culture experiment, each independent experiment consists of three technical replicates). *A one sample t-test was used to compare EHD2-scTNF_R2_ treatment to PBS (*p < 0.05)*

**
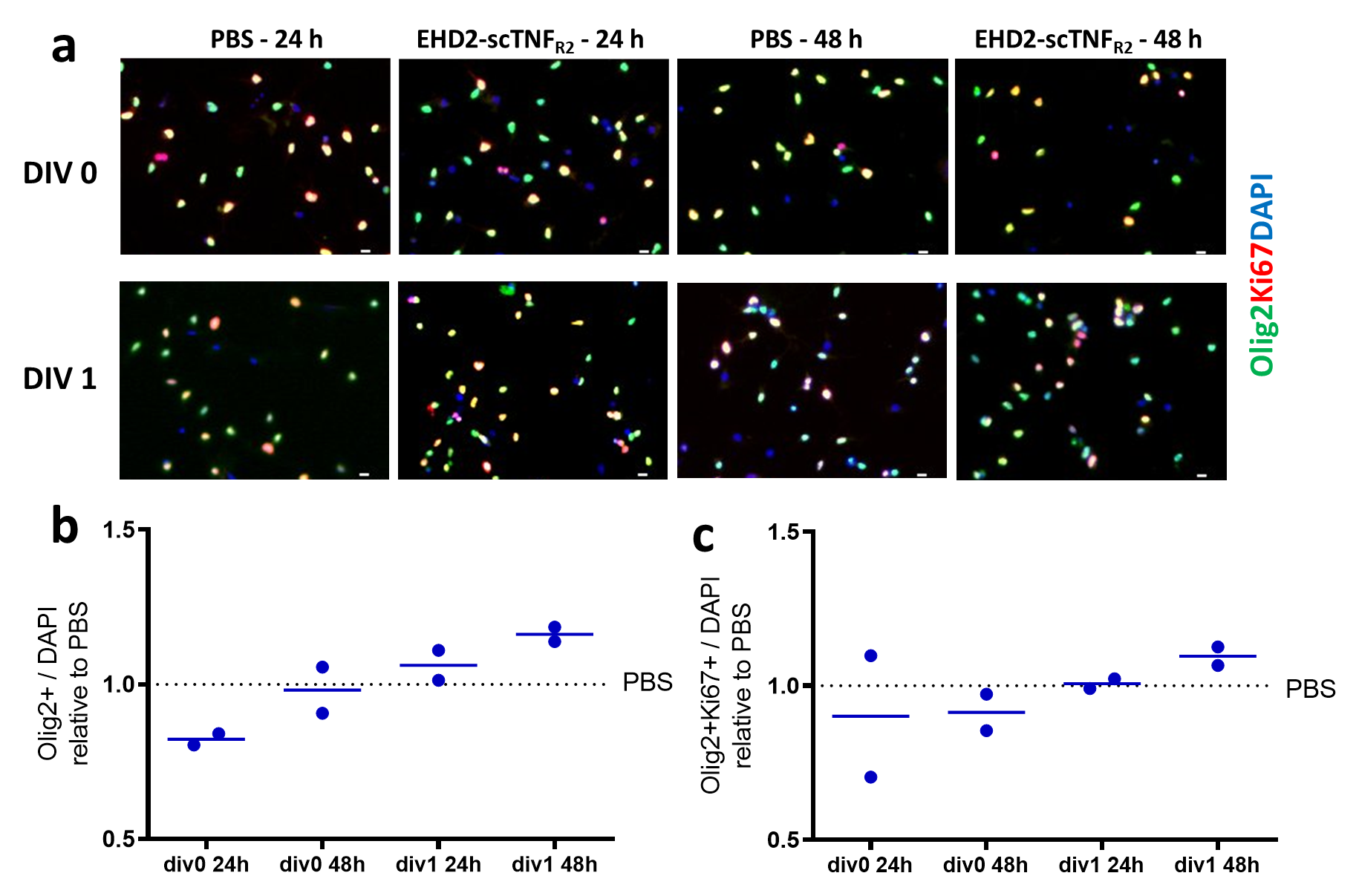
**

**Fig. S7** EHD2-scTNF_R2_ treatment does not alter oligodendrocyte progenitor cell (OPC) proliferation in vitro. **a** Representative image of primary OPC isolated from hu/m TNFR2-ki mice (P1-3) following the experimental set up shown in Fig. S6a and stained for the oligodendrocyte lineage marker Olig2 (green) and proliferation marker Ki67 (red) after 24- or 48 h treatment with PBS or EHD2-scTNF_R2_ [100 ng/ml] starting at 0 or 1 day in vitro (DIV). Nuclei are visualized with DAPI (blue) (**b**,**c**), Olig2-positive cells of total cells (PBS mean ± SEM: div0 24 h = 71.5% ± 4.5; div0 48 h = 66.4% ± 5; div 1 24 h = 66.3% ± 1.7; div1 48 h = 72.7% ± 2.6; **b**) and Ki67-positive cells of Olig2-positive cells (PBS mean ± SEM: div0 24 h = 57.2% ± 4.4; div0 48.h = 66.2% ± 4.3; div 1 24 h = 62.8% ± 4.5; div1 48 h = 56.4% ± 7.5; **c**) were manually counted and data is presented as mean value of two independent cell culture experiments relative to PBS (horizontal line, set at 1 in each independent cell culture experiment, each independent experiment consists of three technical replicates). *A one-sample t-test was used to compare EHD2-scTNF_R2_ treatment to PBS (not significant). Scale bars are 10 μm.* ***a*** *was created with vectors from bioicoins.com*

**Table S3** Summarizing results of manual analysis of ultrastructural features of myelinated axons^a^

|  | **inner area**  **(µm^2^)** | | **myelin thickness**  **(µm)** | | **diameter**  **(µm)** | | **fiber diameter**  **(µm)** | | **g-ratio** | |
| --- | --- | --- | --- | --- | --- | --- | --- | --- | --- | --- |
|  | *mean* | *SEM* | *mean* | *SEM* | *mean* | *SEM* | *mean* | *SEM* | *mean* | *SEM* |
| **PBS – 7 DPT** | **.436** | .024 | **.075** | .007 | **.709** | .021 | **.858** | .017 | **.836** | .016 |
| **EHD2-scTNF_R2_ – 7 DPT** | **.355** | .023 | **.070** | .005 | **.642** | .021 | **.781 *** | .025 | **.832** | .008 |
| **PBS – 14 DPT** | **.459** | .036 | **.089** | .004 | **.725** | .024 | **.904** | .025 | **.808** | .009 |
| **EHD2-scTNF_R2_ – 14 DPT** | **.414** | .035 | **.062** | .009 | **.698** | .036 | **.823** | .046 | **.849** | .016 |

^a^ At least 100 myelinated axons/mouse treated with saline (PBS) or EHD2-scTNF_R2_ were analyzed at 7- and 14-days post treatment (DPT). *n = 3-4 animals/group.* *Unpaired t-test to compare between two treatments at the same time point (*p < 0.05) or two time points of the same treatment (not significant)*
